# How Comparable are Microbial Electrochemical Systems around the Globe? An Electrochemical and Microbiological Cross‐Laboratory Study

**DOI:** 10.1002/cssc.202100294

**Published:** 2021-05-05

**Authors:** Carlo Santoro, Sofia Babanova, Pierangela Cristiani, Kateryna Artyushkova, Plamen Atanassov, Alain Bergel, Orianna Bretschger, Robert K. Brown, Kayla Carpenter, Alessandra Colombo, Rachel Cortese, Benjamin Erable, Falk Harnisch, Mounika Kodali, Sujal Phadke, Sebastian Riedl, Luis F. M. Rosa, Uwe Schröder

**Affiliations:** ^1^ Department of Material Science University of Milan Bicocca U5 Via Cozzi 55 Milan 20125 Italy; ^2^ Aquacycl LLC 2180 Chablis Court, Suite 102 Escondido CA 92029 USA; ^3^ Department of Sustainable Development and Energy Resources Ricerca sul Sistema Energetico S.p.A. Via Rubattino 54 Milan 20134 Italy; ^4^ Physical Electronics 18725 Lake Drive East Chanhassen, Minnesota 55317 USA; ^5^ Department of Chemical & Biomolecular Engineering National Fuel Cell Research Center (NFCRC) University of California Irvine CA 92697 USA; ^6^ Laboratoire de Génie Chimique Université de Toulouse, CNRS-INPT-UPS 4 allée Emile Monso 31432 Toulouse France; ^7^ Institute of Environmental and Sustainable Chemistry Technische Universität Braunschweig Hagenring 30 38106 Braunschweig Germany; ^8^ J. Craig Venter Institute 4120 Capricorn Lane La Jolla CA 92037 USA; ^9^ Department of Chemistry Università degli Studi di Milano Via Golgi 19 Milan 20133 Italy; ^10^ Department of Environmental Microbiology Helmholtz-Centre for Environmental Research – UFZ Permoserstr. 15 04318 Leipzig Germany

**Keywords:** collaboration, fuel cells, electrochemistry, microbiology, statistical analysis

## Abstract

A cross‐laboratory study on microbial fuel cells (MFC) which involved different institutions around the world is presented. The study aims to assess the development of autochthone microbial pools enriched from domestic wastewater, cultivated in identical single‐chamber MFCs, operated in the same way, thereby approaching the idea of developing common standards for MFCs. The MFCs are inoculated with domestic wastewater in different geographic locations. The acclimation stage and, consequently, the startup time are longer or shorter depending on the inoculum, but all MFCs reach similar maximum power outputs (55±22 μW cm^−2^) and COD removal efficiencies (87±9 %), despite the diversity of the bacterial communities. It is inferred that the MFC performance starts when the syntrophic interaction of fermentative and electrogenic bacteria stabilizes under anaerobic conditions at the anode. The generated power is mostly limited by electrolytic conductivity, electrode overpotentials, and an unbalanced external resistance. The enriched microbial consortia, although composed of different bacterial groups, share similar functions both on the anode and the cathode of the different MFCs, resulting in similar electrochemical output.

## Graphical abstract

**The famous five**: A cross‐laboratory study involving five institutions is presented. Each team runs a triplicate identical single chamber microbial fuel cell for 6 weeks varying only the inoculum. Electrochemical and operational parameters are collected. Microbiological analysis is carried out on the influent and effluent and on the electrodes. All the data collected are correlated by using robust statistics.

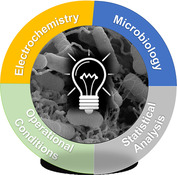

## Introduction

Microbial electrochemical technologies (MET) promise great innovation in different fields, such as environmental pollution remediation, low power generation, biosensing, synthesis of new products and medicine.[^1^, ^2^, ^3^, ^4^, ^5^, ^6^, ^7^, ^8^, ^9^, ^10^, ^11^, ^12^] However, the transfer from laboratory experimentation to field application is still challenging. Mostly, but not exhaustively, these technologies take advantage of a synergistic combination of well‐known electrochemical and microbiological processes, needed to be mastered requires expertise of rarely a common background.^[3]^ In the last decades, the study of microbial fuel cells (MFCs) as a model for primary MET greatly helped in acknowledging the necessity of a common knowledge and methodologies,^[12]^ although the spent efforts were only partially successful.

It has been shown that bacterial cytochromes within the electron transfer chain in biofilms, for example on the anode of an MFC, serve as electron sinks, with pseudocapacitive properties. The power produced by MFCs correlates strongly to the metabolic rate and substrates availability. In turn, it is difficult to maintain a uniform power output over time while operating MFCs, especially if operated in discontinuous or batch mode. Recently, efforts have been made to integrate capacitive materials with MFC electrodes in order to improve power output consistency by adding charge storage capabilities, as has been discussed in recent reviews.[^13^, ^14^, ^15^] This highlights the importance of investigating the possibility of achieving reproducible and constant power output, at rather controlled environmental conditions.

It has to be noted that results obtained among different research groups and even between different scientists in one group, still remain difficult to compare, despite the thousands of studies reported on MFCs.^[16]^ This can be ascribed to the challenge in reproducing the operating conditions[^17^, ^18^, ^19^, ^20^, ^21^] as well to variations in MFC design,[^22^, ^23^, ^24^] electrodes and components,[^25^, ^26^, ^27^, ^28^, ^29^, ^30^, ^31^] inoculum source,[^32^, ^33^] substrate[^32^, ^33^, ^34^] and ways to analyze and express obtained data.[^35^, ^36^, ^37^]

Differences in electrode material and potential, and especially substrate and electrolyte composition can influence the nature of the microbial populations attracted and selected as well as biofilm growth, structure and volume.[^38^, ^39^, ^40^, ^41^, ^42^, ^43^, ^44^, ^45^, ^46^] This in turn affects the energy recovery and removal rates of MFCs.[^38^, ^39^, ^40^, ^41^, ^42^, ^43^, ^44^, ^45^, ^46^, ^47^, ^48^, ^49^, ^50^] Low electrolytic solution conductivity is responsible for low electrochemical output.[^49^, ^50^] Therefore, the solution is often amended with buffer (e. g., carbonate, phosphate, Tris) or electrolyte salts (e. g., KCl, NaCl) to overcome this limitation.[^34^, ^51^, ^52^, ^53^, ^54^, ^55^] The nature, degree of oxidation, biodegradability of the organic substrate also significantly affects the MFC output, with simple organics performing better when compared to more complex organic molecules.[^32^, ^33^] Hence, acetate is the most commonly used model substrate in laboratory studies.[^32^, ^33^]

The precise definition of microbial electroactivity remains open, although now more than 100 “electroactive” microbial species are already recognized.[^56^, ^57^, ^58^, ^59^] For laboratory studies often single, well‐known microbial electroactive species, such as *Geobacter* spp.[^60^, ^61^, ^62^, ^63^] or *Shewanella* spp.,[^64^, ^65^, ^66^, ^67^] fed with simple substrates like acetate or lactate, are used to guarantee improved reproducibility and performance. A more complex panorama is faced within mixed microbial pool and complex or multi substrates, as in waste streams. Natural biofilms are a dynamic complex microbial ecosystem that continuously evolves, then stabilizes on almost all type of solid materials (conductive and non‐conductive), adapting to natural, or engineering, environmental variations and contrasting possible biocide effects. The wide variety of strategies adopted by bacterial communities to survive make biofilms the most advanced form of life on Earth.^[68]^ Typically, electroactive and fermentative bacteria growing on electrodes together have positive synergistic activity, as fermenters can break‐down and consume the complex substrates producing the volatile fatty acids – again most prominently acetate – accessible to electroactive bacteria,[^34^, ^56^] which oxidize this to CO_2_, preventing limiting product accumulation for the fermentative bacteria.

In studies on MFC, power and current generation, chemical oxygen demand (COD) removal and Coulombic efficiency (CE) are commonly reported.[^69^, ^70^, ^71^, ^72^] COD removal and CE seems to be straightforward to report, especially when a known substrate that is source of electrons and carbon (e. g., acetate) is used.[^69^, ^70^, ^71^, ^72^]

Different interpretations are often used for reporting current and/or power. These are normalized to the projected electrodes surface area (anode or cathode), electrode volume, or MFCs’ volume (anodic/cathodic compartment).[^35^, ^36^, ^37^] Interesting similarities as well as differences were underlined from investigations performed in different geographic areas. Hence, the continuous increase of the number of studied systems makes it difficult to determine the influence of a single parameter from a multitude of variable factors.

Larrosa et al. described the need to increase the replications in test with MFCs, while considering the low repeatability of the operating condition.^[73]^ Then, Yang et al. compared MFC performance obtained by a single laboratory in ten years of tests, underlining a variation of about 15 % in the peak of power curves (*n*=24) operating in identical conditions.^[16]^ The same set up utilized by other laboratories worldwide shown a peak in power density roughly 30 % lower and a variability of 29 % among 10 samples considered.^[16]^ More recently, Mateo et al. reported that the uncertainty on the probability of maximum current can be statistically reduced to less than 5 % by operating more than 100 MFC simultaneously under the same conditions.^[74]^

Addressing the need of simple and reproducible methods/standards to approach in general microbial electrochemical systems and specifically MFC, five different institutions from four different countries in US and Europe participated in the here presented cross‐laboratory study. The different institutions strictly followed an agreed protocol for running, testing and evaluating the influence of the microbial composition of local domestic wastewater, as the source of inoculum, on the MFCs key characteristics and performance (voltage over an external resistance, power and current production, COD removal, pH, CE and microbial population dynamic).

All data in this study are represented with their median ($\overset{↔}{x})$ and expanded uncertainty (*U*_exp_) in the form $\overset{↔}{x}\pm U_{exp}$ . Note that *U*_exp_, at confidence level of approximately 95 %, is numerically two times larger than the commonly used standard deviation, that when used alone provides a confidence level of only 68 %. This has to be taken into account when current data is compared with prior studies.

## Results

### Voltage trend

**Figure 1:**
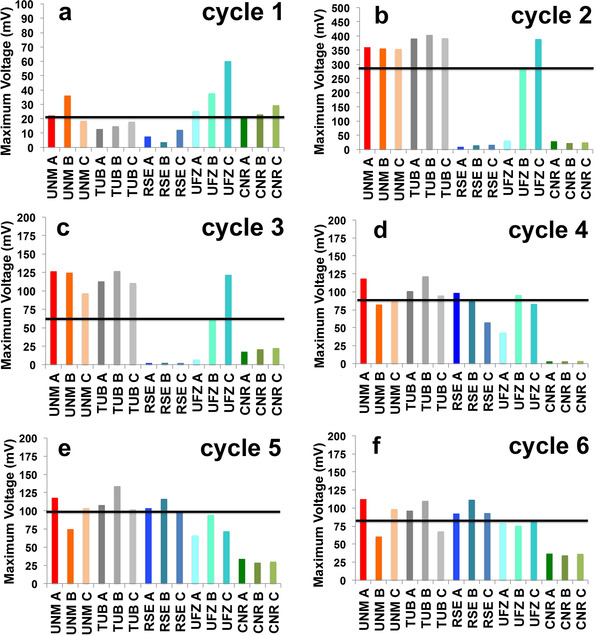
The maximum voltage reached for MFCs at all institutions during cycle 1 (a), cycle 2 (b), cycle 3 (c), cycle 4 (d), cycle 5 (e), and cycle 6 (f). Black line corresponds to the median value.

Voltage trends of the MFCs and the median along with the expanded uncertainty (*U*_exp_) of the voltage at any given time point for the different research institutions are given in the Supporting Information (Figures S1 and S2). The maximum voltage (*V*_max_) achieved during the three phases of the experimentation, for each MFC, is shown in Figure 1. Cycles 1 and 2 indicate a pronounced influence of the raw wastewater source during the acclimation phase (Figure 1a,b).

Table 1 summarizes the maximum voltages (*V*_max_) generated during each cycle along with the corresponding normalized median of absolute deviations (MADN) and expanded uncertainties (*U*_exp_), for the different institutions.

**Table 1:** Maximum generated voltage for each institution accompanied with MADN and expanded uncertainty.

**Acclimation**	**Cycle 1**				**Cycle 2**			
	*V*_max_	MADN	*U*_exp_	*U*_exp_	*V*_max_	MADN	*U*_exp_	*U*_exp_
Institution	[mV]		[%]	[mV]	[mV]		[%]	[mV]
UNM	22	5	48	11	356	3	2	6
TUB	15	3	39	6	392	2	1	3
RSE	8	3	152	12	15	3	35	5
UFZ	38	18	97	36	286	136	95	272
CNR	23	3	29	7	25	3	20	5
*U*_exp_* [%]	120				112

**Stabilization**	**Cycle 3**				**Cycle 4**			
	*V*_max_	MADN	*U*_exp_	*U*_exp_	*V*_max_	MADN	*U*_exp_	*U*_exp_
Institution	[mV]		[%]	[mV]	[mV]		[%]	[mV]
UNM	125	3	5	6	88	8	19	16
TUB	113	3	6	7	101	9	18	18
RSE	2	0	40	1	90	13	29	26
UFZ	62	49	158	97	83	19	45	37
CNR	21	3	24	5	3	0	9	0
*U*_exp_* [%]	293				32

**Steady State**	**Cycle 5**				**Cycle 6**			
	*V*_max_	MADN	*U*_exp_	*U*_exp_	*V*_max_	MADN	*U*_exp_	*U*_exp_
Institution	[mV]		[%]	[mV]	[mV]		[%]	[mV]
UNM	104	21	42	43	99	20	41	41
TUB	108	9	16	17	96	20	42	41
RSE	104	7	13	14	93	1	2	14
UFZ	72	8	23	16	79	6	14	11
CNR	30	2	12	3	36	0	1	4
*U*_exp_* [%]	40				52

The startup time for the MFCs run at UNM, TUB and UFZ were fast, approximately one cycle (Figure S1) and at cycle 2 there was no statistical difference between maximum voltages obtained by TUB, UNM and UFZ. On the contrary, the voltage produced by RSE‐MFCs and CNR‐MFCs was negligible, or very low, during the two‐cycle acclimation period. Adverse meteorological conditions occurred in the period before the sampling at the wastewater treatment plant (WWTP). Heavy rainfall in Milan and in Toulouse caused an exceptional dilution of the raw wastewater, with the consequent decrease of the microbial load and a probable increase of dissolved oxygen in the sampled wastewater of RSE and CNR.

During the acclimation phase, TUB‐MFCs generated the highest voltages of 392±3 mV, followed by UNM‐MFCs (385±7 mV), UFZ‐MFCs (286±272 mV). TUB‐, UNM‐ and UFZ‐MFCs demonstrated significantly higher (*p*=0.05) voltage in this phase than CNR‐ and RSE‐MFCs, where CNR‐MFCs generated statistically higher (*p*=0.05) voltage in comparison to RSE‐MFCs.

A deviation in the protocol was done by RSE team that collected fresh wastewater from their source at the same WWTP for the start of cycle 4. Importantly, RSE‐MFCs started to produce statistically significant voltage (cycle 4), just after the re‐inoculation with the fresh source that this time was without dilution and increased oxygen intake in the wastewater.

Interestingly, CNR had strictly followed the protocol without changing the initial inoculum source. It was expected that due to the diluted inoculum source, the startup time was going to be longer. This was not the case for CNR‐MFCs as no notable voltage was produced during the entire experimentation of six weeks (Figures S1 and S2).

Once the resistance value was decreased from 1000 Ω to 100 Ω (stabilization phase, cycles 3 and 4), the majority of MFCs, except CNR‐MFCs, stabilized the voltage around 100 mV, generating each one a current of approximately 1 mA (ca. 350 μA cm^−2^; Figure 1c–e). During the steady‐state phase (cycles 5 and 6), an increase of the data uniformity and a general decrease of the maximum voltage can be noticed. Indeed, no statistical difference in terms of recorded voltage was observed between all institutions during the cycle 6, except CNR which MFCs had a significantly lower (*p*=0.05) voltage (Figure 1f).

### Polarization and power curves

**Figure 2:**
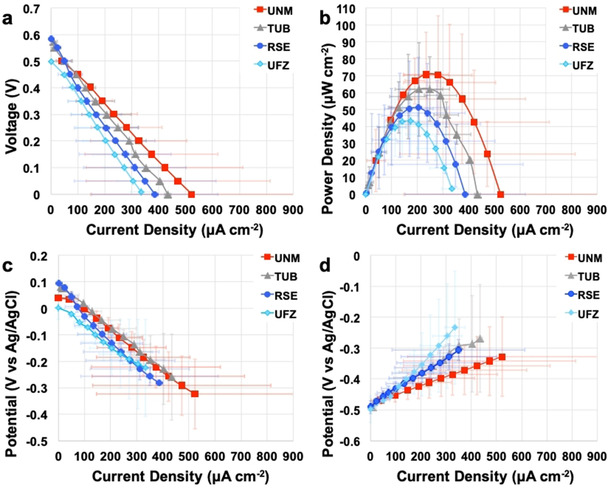
Cell polarization (a) and power curves (b) and cathode (c) and anode (d) polarization curves carried out during cycle 5. Each point is a median of three replicates and the error bars are the corresponding expanded uncertainties.

**Figure 3:**
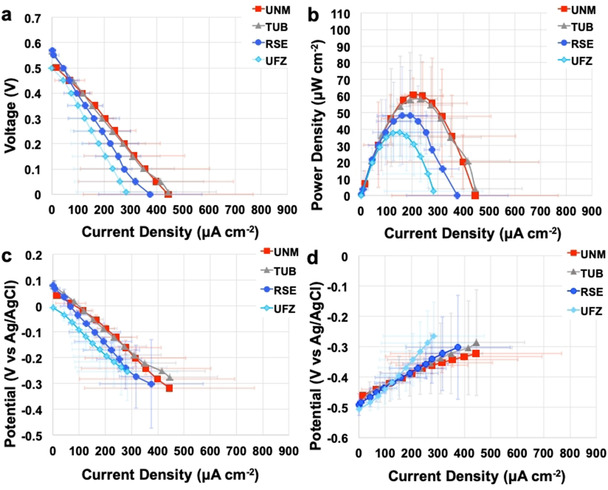
MFC polarization (a) and power curves (b) and cathode (c) and anode (d) polarization curves carried out during cycle 6. Each point is a median of three replicates and the error bars are the corresponding expanded uncertainties.

During the steady‐state phase (cycles 5 and 6), the MFCs were disconnected from the 100 Ω resistances, and polarization curves were performed with the exception of CNR‐MFCs, which did not produce notable voltage. The polarization and power curves for each MFC replicate are presented in the Supporting Information (Figures S3–S10). Figures 2 and 3 show the polarization and power curves and Tables 2 and 3 summarize the main parameters. Each measurement point is represented as a median of the three replicates of the given institution and the corresponding *U*_exp_.

**Table 2:** Summary of parameters extracted from polarization measurements of cycle 5.

Institution	OCV	*J*_max_	*P*_max_	OCP_a_	OCP_c_	*η*a	*η*c	*R*_int_
	[V]	[μA cm^−2^]	[μW cm^−2^]	[mV vs Ag/AgCl]		[mV vs Ag/AgCl]		[Ω]
UNM	540±24	524±377	71±36	−493±30	25±68	210±25	326±1	422±101
TUB	573±36	433±12	62±18	−492±15	79±6	202±111	336±118	354±122
RSE	576±44	385±36	51±12	−489±18	91±3	212±13	373±29	429±92
UFZ	499±0	334±42	43±21	−497±24	1±44	263±242	225±240	223±113
All	547±83	398±132	55±22	−493±15	57±104	213±161	354±99	357±233
*U*_exp_^[a]^ [%]	15	33	41	3	184	76	28	65

[a] For all MFCs.

**Table 3:** Summary of parameters extracted from polarization measurements of cycle 6.

Institution	OCV	*J*_max_	*P*_max_	OCP_a_	OCP_c_	*η*_a_	*η*_c_	*R*_int_
	[V]	[μA cm^−2^]	[μW cm^−2^]	[mV vs Ag/AgCl]		[mV vs Ag/AgCl]		[Ω]
UNM	502±59	444±323	61±18	−477±1	50±1	188±33	335±40	524±572
TUB	565±24	445±30	58±27	−487±1	75±1	190±136	348±186	468±264
RSE	557±56	375±196	48±27	−490±1	78±1	208±149	381±107	130±74
UFZ	500±1	283±193	38±18	−506±2	−1±1	241±73	248±77	234±110
All	524±71	362±259	46±37	−490±4	54±76	203±149	348±96	301±457
*U*_exp_^[a]^ [%]	14	72	81	8	141	73	28	152

[a] For all MFCs.

During cycle 5, the OCV of the MFCs varied between 499 mV and 576 mV with UFZ‐MFCs having the lowest values and RSE‐MFCs demonstrating the highest OCVs (Table 2). The *U*_exp_ of the OCVs varied between 4–8 %, which illustrated a good reproducibility for the given parameter. Among the separate electrodes, the cathode OCP showed low reproducibility especially for UFZ and UNM. On the contrary, the anodic OCP showed a low *U*_exp_ of between 3 and 6 %.

During polarization, the *U*_exp_ of the electrode overpotentials (*η*) for TUB‐ and UFZ‐MFCs was above 40 %. The expanded uncertainty for *J*_max_ was as high as 72 % for UNM‐MFCs and *U*_exp_ for *P*_max_ was 25–50 %. The lack of reproducible MFCs response is in agreement to variations in cathode operation for UNM‐ and RSE‐MFCs and the irreproducibility of the anodes for TUB‐ and UFZ‐MFCs (Figure S5). During cycle 5, the OCV varied in the range of 499 mV and 591 mV and *J*_max_ was between 230 μAcm^−2^ and 655 μA cm^−2^ and *P*_max_ from 29 μW cm^−2^ to 83 μW cm^−2^ (Figure S11a). For most MFCs the cathode appeared to suffer from higher overpotentials limiting the electrode performance (Table 2). The estimated internal resistance during the cycle 5 varied between 183 Ω and 456 Ω.

During cycle 6, the polarization curves (Figure 3) were more dispersed. The expanded uncertainties of *J*_max_ and *P*_max_ were 72 and 81 %, respectively (Table 3). No statistical difference between the different institutions was observed at the level of *p*=0.01 between cycles 5 and 6. OCVs during cycle 6 were similar to OCVs of cycle 5, with values varying between 500 mV and 583 mV. OCV's median was 524±7 mV with an expanded uncertainty of 14 % (Table 3). Higher overpotentials of the cathode (Figure 3c) limited the overall MFC current and power values. *J*_max_ largely spread, with minimum of 218 μA cm^−2^ and maximum of 553 μA cm^−2^. *P*_max_ was between 25 μW cm^−2^ and 67 μW cm^−2^ (Figure S11b). The estimated internal resistance during the cycle 6 varied between 105 Ω and 888 Ω.

### pH trend

The influent of all MFCs was composed of 50 % v/v‐0.1 m carbonate buffer (pH 7.8) and 50 % v/v raw local wastewater. The initial pH of influent solutions for each cycle was 8.3±0.4 with a minimum value of 7.6 and a maximum value of 8.6 (Figure S12a). The pH measured at the end of the weekly cycle varied is shown as an average in Figure S12b and as single value in Figure S13. The pH in the anode compartment decreased around 7.0 at the end of the cycle in several working MFCs, while to increased between 8.5 and 9.0 in some cases. Acidic fermentation could have reinforced the buffer properties of the solution, contrasting the cathode alkalization^[75]^ for the majority of working MFCs. More details about the pH trends over time are given in the Supporting Information.

### COD removal and coulombic efficiency

In most cases higher COD loading in the influent solution correlates with higher COD removal rates,[^76^, ^77^] up to a COD concentration value at which the maximum anodic microbial electrochemical oxidation rate is reached. Therefore, the initial COD loading was kept in a relatively narrow window of high concentration to minimize the influence of COD loading on MFCs operation. The initial COD value, considering all institutions, was 1645±215 mg L^−1^ with *U*_exp_=13 %. Initial and final COD concentrations (along with the corresponding expanded uncertainties) and the COD removal for every MFC replicate are presented in the Supporting Information (Figures S14–S17).

**Figure 4:**
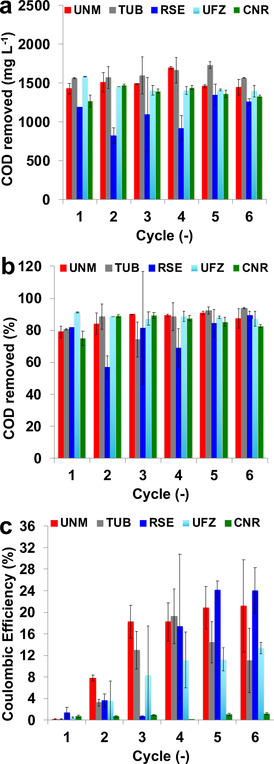
Total COD removal (a), relative COD removal (b) and Coulombic efficiencies (c) for each institution over the 6 successive batch cycles of the cross‐laboratory MFC experiment.

The COD removal per batch over the course of the study and within all replicates was 1430±283 mg L^−1^ with an expanded uncertainty of 20 % among the samples (Figure 4a). The main contributor to the uncertainty is the intra‐laboratory irreproducibility of RSE‐MFCs, which demonstrated a COD removal of 1189±381 mg L^−1^ (*U*_exp_=32 %). The COD removed was 1478±93 mg L^−1^ (*U*_exp_=6 %) for UNM, 1597±135 mg L^−1^ (*U*_exp_=8 %) for TUB, 1380±170 mg L^−1^ (*U*_exp_=2 %) for CNR, and 1424±80 mg L^−1^ (*U*_exp_=6 %) for UFZ. The COD removal was comparable between institutions except for RSE, which received lower initial COD throughout the entire study (Figure S14a).

The relative COD removal was 88±7 % (*U*_exp_=8 %) for UNM, 88±14 % (*U*_exp_=16 %) for TUB, 83±13 % (*U*_exp_=16 %) for CNR, 82±23 % (*U*_exp_=30 %) for RSE and 89±4 % (*U*_exp_=4 %) for UFZ (Figure 4b). The median relative COD removal for all MFCs during the six cycles was 87±10 % with expanded uncertainty of 11 %.

The COD removal was the only parameter in this study that demonstrated good cross‐lab and intra‐lab reproducibility (RSD<25 %). This can be attributed to the predominant role of anaerobic fermentation in the overall organics’ removal and the low impact of the bioelectrochemical conversion on the COD removal. The latter was further confirmed by the low and highly variable CE (Figure 4c).

During the startup phase (i. e., during the first two batch cycles), when bacteria were colonizing the anode at high resistance and developing a biofilm, the CE was generally below 8 %. When the resistance was switched from 1000 Ω to 100 Ω, starting from cycle 3, higher current was obtained (*I*_max_≈1 mA or *J*_max_≈350 μA cm^−2^) and thus higher CE was achieved during the further cycles, except for CNR‐MFCs (Figure 4). The CEs of CNR‐MFCs was always lower than 2 % since the current produced was approximately negligible. For the low performing MFC, the COD degradation is expected to occur due to a mix of aerobic respiration and anaerobic fermentation rather than electrogenesis. The CE values corresponding to each single MFC are presented separately in the Supporting Information (Figure S16).

The low CE values indicate that aerobic transformation and anaerobic fermentation phenomena strongly competed with electrogenesis in consuming the fuel (COD). Different availability of COD could have played a relevant role in the form of concentration (mass transport) overpotential, destabilizing the generated current.^[78]^ Accordingly, CE greatly varied among the institution's triplicates, but also among institutions (Figures 4c and S18). However, the COD removal had a low uncertainty (high stability) for almost all the MFCs, along with the entire study (acclimation, stabilization and steady state), due to the low CE. Just the exceptional high intra‐laboratory irreproducibility of RSE‐MFCs could be related to a low COD (Figure S14a). On the other hand, if the relative COD removal is estimated, the MFCs of the different institutions show statistically identical COD removal including RSE‐MFCs (*p*=0.05). The stabilization of electroactive community structure in the anodic and cathodic biofilm, which are responsible for CE,^[69]^ mostly would have determined the electrochemical performance of each MFC.

### Microbial community structure

The microbial populations of the raw wastewaters (Figure S19), MFC influents (Figure S20), and MFC effluents (Figure S21) were determined for each cycle at the order level. Also, the microbial biofilm communities sampled on the anodes (Figure S22) and cathodes (Figure S23) were investigated at the end of cycle 6 (day 42) for each MFC, except CNR‐MFC, as these did not show a microbial electrochemical performance. Relative abundance of orders of bacteria detected in the raw wastewater is shown in Table 4. The most abundant orders of bacteria detected for the 12 MFCs and in the different components (influent, effluent, anodes, cathodes) at the end of the cycle 6, are summarized in Table 5.

**Table 4:** Relative abundance of bacterial orders (more than 1 % OTU relative abundance) in the raw wastewater. In bold the most abundant orders (more than 10 % OTU relative abundance).

	Raw Wastewater				Total	%
Order	TUB	UFZ	RSE	UNM		
**Clostridiales**	3709	9045	18	1263	14035	30.63
**Pseudomonadales**	406	1765	4275	1349	7795	17.01
**Lactobacillales**	2045	2360	26	427	4858	10.60
Fusobacteriales	1777	1804	4	291	3876	8.46
Burkholderiales	715	962	186	755	2618	5.71
Enterobacteriales	339	707	19	515	1580	3.45
Campylobacterales	40	343	2	806	1191	2.60
Flavobacteriales	24	20	614	482	1140	2.49
Bacteroidales	370	107	2	556	1035	2.26
Synergistales	84	358	1	32	475	1.04
Erysipelotrichales	113	298	0	17	428	0.93
Desulfovibrionales	56	123	0	105	284	0.62
Rhodocyclales	52	84	3	129	268	0.58
Rhizobiales	94	126	32	15	267	0.58
Xanthomonadales	65	67	39	35	206	0.45
Bacillales	59	12	3	5	79	0.17
Alteromonadales	2	1	0	11	14	0.03
Others	1907	2649	118	993	5667	12.37
Total	11857	20831	5342	7786	45816

**Table 5:** Bacterial orders (more than 1 % relative abundance) in the influent, effluent, anode, and cathode. In bold the most abundant orders (more than 10 % relative abundance).

Influent	Effluent	Anode	Cathode
**Pseudomonadales Clostridiales Bacillales** Campylobacterales Lactobacillales Burkholderiales Bacteroidales Enterobacteriales Fusobacteriales Flavobacteriales Rhodocyclales	**Pseudomonadales Clostridiales Bacteroidales** Burkholderiales Rhodocyclales Synergistales Flavobacteriales Bacillales Campylobacterales Desulfovibrionales Xanthomonadales Alteromonadales Lactobacillales Rhizobiales Erysipelotrichales	**Synergistales Clostridiales Lactobacillales Bacteroidales Burkholderiales** Desulfovibrionales Rhodocyclales Enterobacteriales Xanthomonadales	**Bacteroidales Clostridiales Synergistales** Lactobacillales Rhizobiales Oceanospirillales Burkholderiales Rhodocyclales Desulfovibrionales Erysipelotrichales Pseudomonadales Flavobacteriales Alteromonadales Campylobacterales Xanthomonadales

#### Microbial community structure in the inoculum and the anode chamber

Regardless of the wastewater collection location, the influent contained rich and diverse microbial communities, mostly characterized by the presence of fermentative and facultative anaerobes among the orders of *Clostridiales*, *Lactobacillales*, *Fusobacteriales, Bacteriodales*, *Enterobacteriales*. Aerobic bacteria belonging to the orders of *Burkholderiales*, and *Pseudomonadales* were also found (Figure S20). Differently from all other inocula, the first RSE‐MFCs inoculum, which was used from the cycle 1 to the cycle 3, had a relatively poor microbial pool, with the predominance of aerobic *Pseudomonadales* and facultative aerobes *Bacillales* (Figure S20). After the refreshing of the inoculum, at cycle 4, RSE‐MFC influent community structure varied significantly increasing strongly the microbial burden. Interestingly, also UNM‐MFC inoculum in the fourth week differed from the previous inoculum and this might be due to a mistake of sampling (Figure S20).

The microbial community composition of the influent of TUB‐ and UFZ‐MFCs showed the highest similarity with a prevailing abundance of *Clostridiales*, *Lactobacillales* and *Fusobacteriales* (Figures S19 and S20). Both institutions are located in a similar climate in Germany, and wastewater from WWTP is subject to the same water legislation rules. *Pseudomonadales*, that was the most abundant order in the influent of RSE‐ and UNM‐MFCs (genus *Acinetobacter*), accompanied *Bacillales* in the case of RSE‐MFCs and *Campylobacteriales* (genus *Arcobacter*) for UNM‐MFCs.

The effluent samples showed microbial community structures consistent with the corresponding influent and inoculum communities (Table 5, Figure S21). *Clostridiales* were highly abundant in the effluent of TUB‐, UFZ‐ and UNM‐MFCs. TUB, UFZ and UNM effluents were also characterized by the co‐occurrence of *Bacteriodales* and *Desulfovibrionales*. The latter appeared to be able to perform microbial extracellular electron transfer.^[79]^

**Figure 5:**
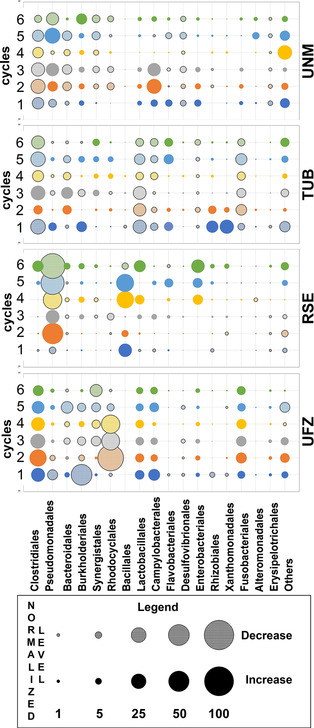
Variation in the bacterial community profiles of effluent samples at order level over batch cycle. The variation in microbial species was evaluated as the difference of the relative abundance of given bacteria between the influent and effluent samples. A median of the three replicates for each institution was taken.

Figure 5 shows the dynamics of the effluent microbial population over time induced by MFCs operation for the different institutions. A median of the three replicates for each cycle was taken to evaluate the difference in the relative abundance of each bacterial order between influent and effluent samples. TUB‐MFCs revealed a significant decrease in abundance of *Clostridiales*, *Lactobacillales, Fusobacteriales* and *Campylobacteriales*. An increase in *Synergistales*, *Desulfovibrionales* and *Flavobacteriales* species was also observed. Based on the similarities between the influent and effluent microbial community of TUB‐ and UFZ‐MFCs, it was expected that the induced changes in microbial species abundance for UFZ‐MFC would be similar to TUB‐MFCs. Interestingly, that was not the case and an opposite trend in microbial dynamics was observed for UFZ‐MFC, with an increase in the abundance of *Clostridiales*, *Lactobacillales*, *Fusobacteriales* and *Campylobacteriales* and a decrease in *Synergistales*, *Rhodocyclales* and *Desulfovibrionales* (Figure 5).

A decreased abundance in *Synergistales*, and *Desulfovibrionales* along with *Clostridiales* and *Bacteriodales* was characteristic for UNM‐MFCs. This was accompanied by an increase in *Burkholderiales*, *Lactobacillales* and *Campylobacteriales* species. Thus, the changes in the microbial community during MFC operation were more similar between UFZ‐ and UNM‐MFCs rather than UFZ‐ and TUB‐MFCs. RSE‐MFCs demonstrated an improved electrochemical performance towards the end of the study when the inoculum was changed and a decrease in aerobic *Pseudomonadales* was observed, concomitant with an overall increase in *Bacillales*, *Enterobacteriales*, *Lactobacilles* and *Burkholderiales*.

#### Microbial community structure at the anodes

The predominant bacterial populations of the anode‐associated bacteria were really specific for each institution. Order‐level and family‐level taxonomic distribution of 16S rRNA community profiles for anodic electrochemically active biofilms are shown in Figure S22a and S22b, respectively. UNM‐MFCs, characterized with the highest MFC power output and with a high variability between the MFCs showed, however a similar community among the triplicate anodes, mostly composed of *Synergistrales* (34–61 %), *Clostridiales* (19–24 %) and *Bacteroidales* (11–22 %). TUB‐MFCs anodes were populated by lower percentage of *Synergistrales* (25–31 %), as well as by *Clostridiales* (16–18 %), but similar for *Bacteroidales* (10–31 %) and higher for *Desulfovibrionales* (5–10 %) in comparison to UNM‐MFCs, where *Desulfovibrionales* were only 3 % of the anodic community. UFZ‐MFCs anodes were characterized with the prevalence of *Burkholderiales* (10–50 %). Almost exclusively *Lactobacillales* dominated the anodic community of RSE‐MFCs. On the contrary, *Lactobacillales* were not enriched in the anodic biofilms of TUB‐ and UFZ‐MFCs, although their relevant presence in the influents. *Lactobacillales* enrichment on electrodes from mixed bacteria population are seldom mentioned, but several examples of pure cultures of *Lactobacillus* have been positively exploited in MFCs.[^80^, ^81^, ^82^]

**Figure 6:**
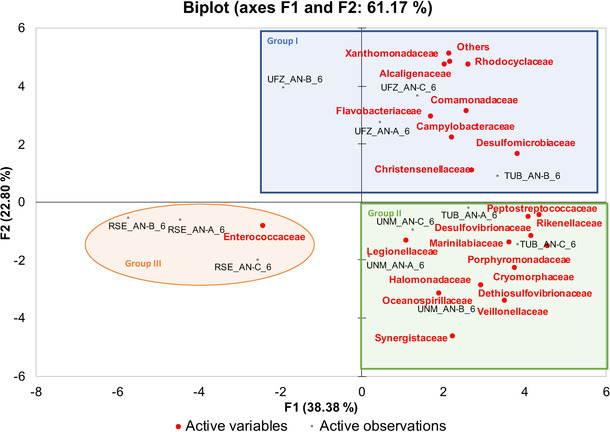
PCA biplot of where scores (observations) and loadings (variables) for MFCs with different microbial species populating the anode surface are plotted on the first two components. Only variables extracted from the microbial community analyses of the anodes at family‐level are included in the biplot.

PCA analysis of the anodic communities of the 12 MFCs from the different institutions was performed at the family‐level to further look into similarities and differences between anodic biofilms (Figure 6). A clear separation in three major groups was revealed. Group I encompassed only the anodes of UFZ‐MFCs. They were characterized with the presence of *Alcaligenaceae*, *Comamonadaceae*, *Dethiosulfovibrionaceae*, *Rhodocyclaceae* and *Desulfomicrobiaceae*. TUB‐ and UNM‐MFC anodes were clustered together in Group II, which demonstrated high versatility, with predominance of *Dethiosulfovibrionaceae, Synergistaceae, Porphyromonadaceae* and *Desulfovibrionaceae*. RSE anodic community deviated significantly from all other institutions with *Enterococcaceae* populating up to 90 % of the anodic community for RSE‐MFC‐B. *Desulfovibrionaceae* species showed abundance of approximately 3 % from the overall microbial population for TUB‐MFCs, 0.5 % for UFZ‐MFCs, 0.1 % for RSE‐MFCs and 0.4 % for UNM‐MFCs. *Desulfovibrionaceae* and *Desulfomicrobiaceae* which have been shown capable of extracellular electron transfer at the anode of MFCs^[83]^ were the only *Deltaproteobacteria* species found in the current study. Species from genera *Desulfovibrio* and *Desulfomicrobium* are well known as ubiquitous sulfate reducing bacteria.[^84^, ^85^, ^86^]

#### Microbial community structure over the cathode electrodes

*Bacteroidales* and *Clostridiales*, strictly anaerobes[^87^, ^88^] were the predominant bacterial orders highlighted at the cathode of all MFCs regardless of the institution and the replicates (Figure S23a). No major differences in microbial population between cathodes at order‐level were noted. The abundance and predominance of given bacteria is highly dictated by the influent microbial structure. A large number of bacteria families populated the cathodes as reported in Figure S23b. Almost one hundred accounted to only 50 % of the total number, with less than twenty reaching more than 3 % of the total OTUs and just *Rikenellaceae* accounted for more than 10 %. Few bacteria genera were clearly classified. Among the others, *Enterococcus* and *Nitricola* prevailed significantly over the others, overcoming the 5 % of total OTUs in some cathodes.

## Discussion

### The role of the inoculum in developing bioelectrodes

The results of the microbiological analyses clearly show the presence of a rich and diverse microbial community, including anaerobic, fermentative and (at least) facultative anaerobes. The anoxic conditions are most important for developing an electroactive bacterial community in MFCs.

The unfeasibility of building a performing MFC by a pool overly rich of aerobic bacteria was well evidenced with the underperforming RSE‐MFCs, which inoculum were characterized by a microbial pool composed of almost sole aerobic *Pseudomonadales* and facultative aerobes *Bacillales* (Figures 5 and S21). The aerobic metabolism of these bacteria supported the fast COD removal in these MFCs, that was similar to the others, however without microbial electrochemical activity. When the inoculum of RSE‐MFCs was refreshed with an inoculum being more consistent and richer of anaerobic bacteria, the stabilization of a well performing anodic biofilm occurred, persisting towards the end of the operation. The anodic community of performing RSE‐MFCs, which deviated significantly from all other institutions, enriched almost exclusively by *Lactobacillales*, with *Enterococcaceae*, populating up to 90 % for RSE‐MFC‐B. These bacteria develop in slightly acidic conditions (pH 5.5–6.5) and sometimes even supports more acidic conditions (pH>3.5).[^89^, ^90^, ^91^] Looking at the influent and effluent data of pH for RSE, it can be seen that pH was much more acidic in the last cycles (Figures S12 and S13) and that in general the bulk pH was the most acidic among the WW from the 5 institutions. This condition can significantly contribute to lower the internal resistance of the MFC (which approached the external one) during the last two cycles, when these MFCs showed an identical performance to other MFCs.

The dynamics of the microbial population on the anodes and the cathodes (Figures 6, S21, S22, and S23), which had unique and complex compositions (Figures S22 and S23) for each institution, confirm and add more specific insight about the role of the inoculum. Notably, the best performing of UFZ‐MFC was the only one with the anode dominated by *Burkholderiales* (50 %) and *Xantomonodales*, which both are strict aerobes already found in electroactive pools.^[92]^ They are large fermentative communities of bacteria that most likely live very well in association with the anaerobic electroactive biofilm at the anode, facing to the bulk, where oxygen bioavailability is possible, if the anolyte is not completely anaerobic. Their possible synergistic role towards the anaerobic microbial composition (also detected in the pool, but in minor percentages) is to rapidly deplete oxygen that is incoming towards the anode. The strict anaerobic condition of the anode, allowing MFC to perform, was documented by the low anodic potential of this UFZ‐MFC, similar to the others. In the case of CNR‐MFCs, the poor bacteria pool of the inoculum (unchanged during the study) totally contrasted the developing of anaerobiosis at the anode. The bacteria burden of the inoculum is a key parameter for MFC operations. Interestingly, if the starting period works badly because of a poor inoculum, then the MFC does not start to produce electricity.

More critical and complex is the analysis of the cathodic biofilm performance. As a general thought, the same bacteria groups predominating the anodic biofilms were also found on the cathodes, although in richer bacterial pool and different relative abundance (Figure S23). This result is due to the lack of physical separator (i. e., a membrane or separator between the cathode and the anolyte).[^93^, ^94^] It can be underlined that the porous cathode, which one side is exposed to the air and the other side interfacing the anaerobic anolyte, presents a larger gradient of conditions than the anode, being ecological niches for a multitude of different microorganisms. Bacteria within the biocathode can perform, large number of redox reactions, such as aerobic, microaerophilic and anaerobic pathways that simultaneously contribute to the overall electron transfer from the conductor to O_2_ ^[95]^ or provide an alternative reduction pathway (e. g., sulfate, nitrate).[^96^, ^97^] This fact explains why the majority of bacterial species found on the cathode exposed to the air are obligate or facultative anaerobes (the contrary on the anode). The difference of electrode potentials applies divergent selection pressure and specific growth rates on the microbial biofilm communities, further driving unbalances in the relative abundance of dominant communities on the electrodes.[^98^, ^99^, ^100^]

*Clostridiales*, which reached more than 20 % of relative abundance in the air breathing biocathodes here as in other works,[^101^, ^102^] with *Bacteroidales (*one of the most represented taxa also in bioanodes from activated sludge),^[103]^ guarantee the presence of anaerobic conditions at the cathode surface, in spite exposure to air.^[104]^ Cathodes, on the other hand, were significantly populated by facultative anaerobes, such as *Rhizobiales*, *Rhodocyclales* and *Alteromonadales*.

*Pseudomonodales*, which characterized the influent of RSE and TUB, were the sole aerobic bacteria that enriched over all the cathodes (Figure S23). This bacterial order has been previously evidenced on the surface of gas‐diffusion biocathodes.[^104^, ^105^] *Rhodocyclales* species, also well represented in the cathodic microbial pools, are characterized by their high tolerance to oxygen.^[106]^ A special attention deserves the *Oceanospirillales* order and particularly the genus *Nitricola* and *Halomonas* that was significantly present in the cathodic community, with up to 10 % relative abundance, among a large group of denitrifying bacteria. Both these genera are capable of using O_2_ or NO_2_^−^ as electron acceptors.[^107^, ^108^] *Nitricola* has been previously found to strongly predominate the microbial community of biocathodes using swine manure as inoculum.^[109]^ *Halomonas* was enriched significantly at the biocathodes in a previous study,^[104]^ where it was quantified how an anaerobic biocathode can perform better than more aerobic ones in air‐breathing MFCs. Hence, the stabilization of anaerobic communities able to transfer electrons faster through a chain of mediators can confirm the mechanism sustaining (but also limiting) the cathodic reaction. The strict anaerobic metabolism, rather than the direct reaction with oxygen (scarcely diffusing through the biofilm) enhance the biocathode.

The bacteria involved in the sulfur cycle (as well in the nitrogen cycle), such as *Desulfovibrionales* which (as well as the anode) enriched in the cathodes of this study and commonly detected on MFC electrodes,[^83^, ^110^] typically can play this role, producing sulfides. This type of sulfate reducing bacteria is well known to enhance the cathodic reaction in anaerobic environment also in the case of corrosion, where metals, instead fuel are oxidized.^[111]^ Moreover, *Desulfovibrionales* can use hydrogen as the sole energy source and acetate and CO_2_ as carbon source.^[112]^ Several strains of this type of bacteria are also able to use nitrate as alternative electron acceptor and hydrogen as fuel.^[113]^

The concentration of mediators produced by the different (anaerobe or micro‐aerophile) bacterial consortium at the cathodes, and the diffusion rate of mediators through the thick cathodic biofilm to the oxygen of the air, in conclusion, could determine the large part of variability of the MFC performance. These aspects, as well as the syntrophic microbial activity of fermenters and electrogens, deserve further investigation, aiming to better standardize and improve the MFCs behavior.

### Principal component analysis considering microbial community structure analysis and data of MFC electrical and treatment performances

**Figure 7:**
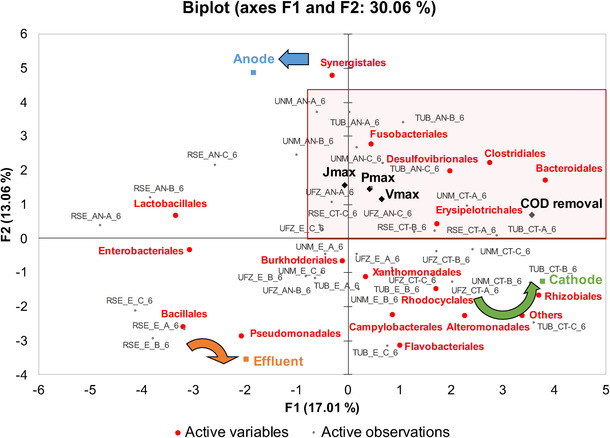
PCA biplot of where scores (observations) and loadings (variables) for MFCs with different microbial species populating the anode and cathode surface as well as effluent samples are plotted on the first two components. Only variables extracted from the last cycle of the experiment are included. *J*_max_ and *P*_max_ are the maximum current and power measured during polarization curves and *V*_max_ is the maximum voltage generated under 100 Ω during cycle 6. COD removal is the total COD removal for cycle 6.

PCA analysis of the community profiles of the anodes, cathodes and effluent samples, and their correlation with MFCs electrochemical parameters, operational parameters and COD removal was performed (Figure 7).

According to this statistical analysis, the cathodic bacterial community was more similar to the effluent microbial population, while the anodic biofilms were more specific for the different institutions and certainly linked with the origin of the inoculum. The majority of anodic biofilms were highly represented by bacteria from the order *Synergistales* that seldom was reported in MFCs pools.[^114^, ^115^] Nevertheless, several bacterial groups belonging to *Synergistales* order are well known strictly anaerobic microorganisms which synergistically act in dark fermentation,^[116]^ participating in the metabolism of hydrogen and in the production of biogas from sludge digestion[^117^, ^118^] as well to the microbial electrosynthesis of hydrogen.[^118^, ^119^, ^120^]

Higher energy recovery is associated with higher abundance of *Desulfovibrionales* at the anode, and the higher COD removal is clustered with fermentative bacteria such as *Clostridiales* and *Bacteriodales*. Clearly a syntrophic coupling between fermentative and electrogenic populations occurs.[^45^, ^121^, ^122^, ^123^]

All reactors featured a high relative abundance of fermentative bacteria in the influent, effluent and on the anode and cathode. Fermentative bacteria have been reported in many wastewater‐enriched MFC converting sugars and other complex substrates to simple volatile fatty acids that are the preferred carbon sources for anodic electroactive bacteria such as *Desulfovibrionales*.[^109^, ^124^, ^125^, ^126^, ^127^, ^128^, ^129^]

Analyzing the overall data from the electrochemical point of view (voltage, power and current medians), it can be noted that MFCs demonstrate high intra‐laboratory deviation as well as cross‐laboratory deviation to the stationary phase between the three replicates (Table 1). This can be pointed out from stochastic events as previously presented.^[45]^ Nevertheless, no significant differences between the median values of the different institutions was observed at the level of *p*=0.01, including parameters from cycles 5 and 6.

The *U*_exp_ of OCVs varied relatively little, between 4–8 %, indicating a good reproducibility for the given reactor system and operation. The deviation from the median was more pronounced at the beginning of the experiment for UFZ‐MFCs and towards the final cycles for UNM‐MFCs and TUB‐MFCs. It has to be emphasized that there was no uncertainty associated with the startup time for voltage generation of the replicate MFCs of each institution (Figures S1 and S2), remarking the reliability and reproducibility of this parameter.

Higher *U*_exp_ was noticed for *J*_max_ and *P*_max_ (Table 2 and 3). Indeed, *P*_max_ measured during cycles 5 and 6 had an expanded uncertainty of 41 % and 81 % respectively. A relative high dispersion of *P*_max_ data is expected, since both the uncertainties of voltage and current multiply (*P*=*V*×*I*).

Analyzing single electrode OCPs, more variability (also intra‐laboratory) is evidenced for cathodes (between −10 mV and 86 mV vs Ag/AgCl) than for anodes (between −477 and −511 mV vs Ag/AgCl). Accordingly, the anodic OCP showed a low *U*_exp_ quantified between 3 and 6 %, which excludes relevant variation of charge overpotential in productive anodes, in spite the predominant species of the anode‐associated bacteria were specific for each institution (Figure S22). Therefore, the cathodic performance is the critical element limiting, and also destabilizing, the MFC performance. The low surface area of the cathodes (roughly half compared to the anode, 2.90 vs. 6.25 cm^2^) enhanced this effect. For biocathodes, a complete deactivation can occur quickly after the steady‐state was reached, due to the precipitation of carbonates at the interface between biofilm and the electrode surface.[^130^, ^131^, ^132^, ^133^] An initial scaling phenomenon could cause the decreasing of performance detected for cycle 6 compared to cycle 5.

## Conclusions and Perspective

Is it possible to develop common standards for MFC studies across different countries? This work has given a preliminary positive answer to this question providing a validated protocol for measuring MFC performances. The possibility of achieving similar bioelectrochemical results, independently of the location, and inherent variations in the microbial consortium within the same type of inoculum is also demonstrated.

To investigate the influence of the inoculum source on MFC performance, identical MFCs were tested in a cross‐laboratory study involving five different institutions worldwide. These MFCs were inoculated with raw wastewater from local domestic wastewater treatment plants in geographically different locations. After the acclimation stage, despite different initial inocula, all MFCs reached similar power outputs (55±22 μW cm^−2^) with a maximum power density of 83 μW cm^−2^ and a corresponding current stabilizing to a maximum of about 1 mA cm^−2^. Importantly, if the inoculum is very diluted and anaerobiosis is difficult to attain, a change in inoculum is suggested and envisioned. The microbial communities composing the inoculum are a key parameter for MFC startup. COD removal efficiency for 7 days of HRT was 87±9 % (1430±283 mg L^−1^) despite the large microbial diversity. It is believed that the similar performance is a result of the synergistic metabolism in consortia of fermentative and electrogenic bacteria. These consortia, although composed of different bacterial species, share similar function in digesting acetate and the other organic components of dissolved COD. Among them, bacteria from the orders *Clostridiales*, *Bacteroidales*, *Burkholderiales*, *Synergistales*, *Lactobacillales* and *Desulfovibrionales* seem to play a relevant role. Higher energy recovery was associated with higher abundance of *Desulfovibrionales* or *Lactobacillales* at the anode, and higher COD removal was clustered with fermentative bacteria such as *Clostridiales*, *Bacteriodales* and *Synergistrales*.

Owing to the complex nature of anode and cathode biofilms, they are characterized with high uncertainty as previously shown.[^134^, ^135^, ^136^] Based on the results from the less productive cycles, it could be concluded that the MFC startup time is strictly related to the abundance and richness of bacteria in the inoculum, which develop anaerobic conditions. Importantly, the bacteria burden of the inoculum is a key parameter. If anaerobic conditions are not established in the anodic chamber due to a poor and diluted inoculum, the MFC does not produce significant electricity therefore it is suggested to replace the inoculum rather than to wait longer.

The uncertainty of the electrochemical performance in steady state, limited by the chosen external resistance (100 Ω), is mostly influenced by the stability of the microbial cathode. Well‐established electroactive microbial communities and a controlled pH of the anolyte can reduce the overpotentials, which would allow the MFCs to reach optimized performance faster.

More sophisticated and optimized experimental designs need to be envisioned for acquiring deeper insight on the interactions between fermenter and electrogenic bacteria, and their enzymatic electron mediators, targeting to improve the generated power, CE and the reproducibility of the data. This is urgently needed to develop common standards needed for development and implementation of MFC and other MET as technology in our society.

## Experimental Section

### Cross‐Laboratory Study

The involved international research groups were: 1) University of New Mexico (**UNM**) located in Albuquerque‐NM, USA; 2) Ricerca sul Sistema Energetico (**RSE**) and University of Milan, located in Milan, Italy; 3) CNRS, Université de Toulouse (**CNR**), located in Toulouse, France; 4) Technische Universität Braunschweig (**TUB**), located in Braunschweig, Germany; 5) Helmholtz‐Centre for Environmental Research – UFZ (**UFZ**), located in Leipzig, Germany. The names of the MFC for each institution were abbreviated as UNM‐MFC, RSE‐MFC, CNR‐MFC, TUB‐MFC and UFZ‐MFC respectively (Table 6). Each laboratory ran three MFCs in parallel to estimate intra‐laboratory reproducibility. Each replicate MFC belonging to each institution was assigned with the letters A, B and C (Table 6).

**Table 6:** Institution, location and abbreviations used for this current work.

Institution	Abbreviation	Location	MFC #
University of New Mexico	UNM	Albuquerque, NM, USA	UNM‐MFC‐A
			UNM‐MFC‐B
			UNM‐MFC‐C
Ricerca Sul Sistema Energetico	RSE	Milan, Italy	RSE‐MFC‐A
			RSE‐MFC‐B
			RSE‐MFC‐C
CNRS, Université de Toulouse	CNR	Toulouse, France	CNR‐MFC‐A
			CNR‐MFC‐B
			CNR‐MFC‐C
Technische Universität Braunschweig	TUB	Braunschweig, Germany	TUB‐MFC‐A
			TUB‐MFC‐B
			TUB‐MFC‐C
Helmholtz Center for Environmental Research	UFZ	Leipzig, Germany	UFZ‐MFC‐A
			UFZ‐MFC‐B
			UFZ‐MFC‐C

### Cross‐laboratory study design

This work consisted of investigating triplicate identical MFCs using the same reactor design, identical electrodes (geometry and material), materials, working temperature, substrate and form of data collection and reporting. The only variation among the MFCs of the different institutions was the inoculum (fresh local raw domestic wastewater), responsible for 50 % in volume of the MFC reactor. Electrochemical and chemical parameters of interest were measured following pre‐established protocols (see details in Table S1).

The duration of the MFC experiments was 42 days (6×7 days), corresponding to 6 complete batch cycles of solution (carbonate buffer/raw wastewater and 3 g L^−1^ sodium acetate). Each cycle had duration of 7 days and started the same day for all groups. The solution was fully exchanged every cycle with fresh mixture of carbonate buffer/raw wastewater and 3 g L^−1^ sodium acetate.

MFC operation was subdivided into three phases among the 6 feed cycles: 1) Acclimation (cycles 1 and 2); 2) Stabilization (cycles 3 and 4); 3) Steady state (cycles 5 and 6; Table S1).

#### Acclimation phase (cycle 1 and 2)

MFCs were prepared and started the operation the same day when each institution collected the fresh local raw domestic wastewater, from each related local WWTP (Table S2). After the initial inoculation, an external resistance (1000 Ω, Radio Shack, USA) was connected between the anode and cathode.

#### Stabilization phase (cycle 3 and 4)

At the beginning of cycle 3 (day 14), after the solution was renewed, the external resistance was changed from 1000 to 100 Ω. This value was found to be close to the MFC internal resistance based on previous experiments with the same MFC system^[137]^ and it was kept for the rest of the study to test the MFC performance. Please note that TUB and UNM changed the resistance from 1000 to 100 Ω on day 16 (and not on day 14; Table S1). During these cycles, the produced power from each MFC was supposed to rise and stabilize. Few final adjustments in the MFC set occurred in this phase. At cycle 4, the inoculum of the three RSE‐MFCs (unproductive at this phase) was substituted with a fresh one collected from the same WWTP. In the case of CNR‐MFCs, although these MFCs did not show any electrochemical performance, the inoculum was not changed.

#### Steady state phase (cycle 5 and cycle 6)

At the end of cycle 4, the electrochemical behavior of each MFC was supposed to be stable and a final protocol of MFC characterization was carried out for the cycle 5 and cycle 6. Single electrode polarization curves were performed in this phase and power curves were then calculated except for the CNR‐MFCs that were not productive. At the end of the cycle 6, the test ended and the electrodes and their weekly‐collected samples were sent to the J. Craig Venter Institute (La Jolla‐CA, USA), which was in charge for the next generation sequencing. Unfortunately, the samples from CNR‐MFCs were lost during shipment and the sequencing was performed on the samples of the other four institutions only.

The voltage generated over the external resistance of each MFC during the cycles was continuously measured and registered. COD removal and pH of the effluent solution were weekly monitored and CE was calculated. Microbiological analyses of planktonic bacterial communities sampled from influent and effluent MFC solutions, as well as of biofilm bacterial communities sampled from the anode and cathode surfaces of each MFC at the end of each cycle were performed using next generation sequencing.

### Cell assembly and operation

**Figure 8:**
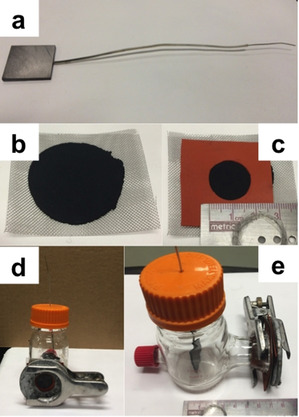
a) Anode; b) cathode; c) gasket on the cathode; d) lateral hole showing the part of the cathode exposed to ambient air; e) lateral view of the built MFC with distance between anode and cathode of 7 cm.

Identical membrane‐less single‐chamber glass MFCs (Arbore Cataldo, Milan, Italy) with an empty volume (electrolyte solution) of 125 mL were used during the experiments. The electrodes were all built identically. The cathodes were manufactured at the UNM and the anodes were manufactured at the UFZ. The anode (Figure 8a) was immersed into the electrolyte solution and kept in a central position within the bottle. The cathode (Figure 8b,c) was screwed on the lateral hole of the glass bottle with one side facing the electrolyte and one side facing the ambient air (Figure 8d). Anode and cathode were kept at a distance of 7 cm (Figure 8e). Triplicate MFCs were run in parallel in batch for the same duration at 25 °C, temperature kept by common laboratory incubators.

### MFC Electrodes

#### Anodes

Graphite plates (2.5×2.5×0.2 cm, CP‐2200 quality, CP‐Handels GmbH, Wachtberg, Germany) attached to a titanium wire (0.5 mm diameter, 99.6 % purity, Goodfellow GmbH, Naunheim, Germany) were used as anodes. All surfaces except the working one (2.5×2.5 cm, 6.25 cm^2^) were covered with insulating epoxy glue (Epoxy HT2, R&G GmbH, Waldenbuch, Germany). The conductive surface was sanded with sandpaper (grit 1000) and washed profusely with MilliQ water. The titanium wire was insulated with Teflon thermoretractable tubing (ABB Shrink‐Kon HSB 46‐C, Germany).

#### Cathodes

Air breathing gas‐diffusion cathodes were used. Activated carbon (AC; Norit SX Plus, Sigma Aldrich) was mixed in a blender with polytetrafluoroethylene (PTFE; 60 wt % emulsion, Sigma Aldrich) and then pressed on a stainless steel mesh (MacMaster, USA) at 1400 psi for 5 min.[^138^, ^139^, ^140^, ^141^, ^142^] No thermal treatment was applied to the cathodes. The AC:PTFE ratio was 80 : 20 % by weight. The cathode area exposed to the solution was 2.9 cm^2^, delimited by the lateral hole of the glassy MFC (Figure 8c).

#### Inoculum

The MFCs were inoculated with a solution composed of 50 % v/v of raw local domestic wastewater and 50 % v/v of 0.1 M carbonate buffer with 0.1 M KCl, pH 7.8. Sodium acetate (3 g L^−1^, 24 mM) was added in the solution as an extra organic substrate. The carbonate buffer with 0.1 M KCl was prepared using: 0.031 g L^−1^ (0.58 mM) ammonium chloride, 7.455 g L^−1^ (100 mM) potassium chloride, 8.064 g L^−1^ (96 mM) sodium hydrogencarbonate, 0.01 g L^−1^ (0.096 mM) sodium carbonate and 0.48 g L^−1^ (4 mM) sodium dihydrogen phosphate. The pH of the carbonate buffer was adjusted to 7.8 using HCl or NaOH. Carbonate buffer was used to increase solution conductivity and attenuate the differences in the conductivity of the wastewater of each laboratory. Each group collected their raw local wastewater from regional wastewater treatment plants (WWTP) reported in Table S2 and conserved the raw wastewater in the fridge at 4 °C. RSE team collected a new local wastewater batch utilized from week 4.

### Electrochemical measurements

The MFC voltage was recorded through a voltage recorder different for every group (Table S2). MFC polarization curves were performed using two independent potentiostats. The first potentiostat was used to carry out the overall polarization curve where the counter electrode channel (short circuited with reference channel) was connected to the air cathode and the working electrode channel was connected to the anode of the MFC. Linear Sweep Voltammetry (LSV) was run from OCV to 10 mV at a scan rate of 0.2 mV s^−1^. The second potentiostat was utilized for monitoring the cathode and anode potentials during the polarization curves in order to discriminate the effect of each electrode. The power (*P*) generated was calculated by using the following equation: P=V×I *(V*=voltage; *I*=current*)*. Power density and current density were reported by normalizing the power and current to the cathode geometric surface area (2.9 cm^2^). Each group utilized different potentiostats and different data log systems as reported in Table S2.

Four common parameters were used to evaluate the MFC performance: 1) maximum voltage (*V*_max_) measured over an external resistance during the 6 batch cycles; 2) open circuit voltage (OCV); 3) maximum short circuit current density (*J*_max_); 4) maximum power density (*P*_max_). The last three parameters were measured during the overall polarization curves. In addition, the open circuit potential (OCP) of the separate electrodes was measured in order to establish which electrode was limiting the cell performance. The maximum electrode overpotential (*η*_a_ and *η*_c_ for the anode and cathode respectively) was used, which was calculated as the difference between the electrode potential at maximum current and the respective OCP ($E_{I_{max}}-\;E_{I_{0}}$ *or* $E_{I_{max}}-\;E_{OCP}$ ). Varying the resistance (external load) between the MFC electrodes, the rate‐limiting electrode will undergo a higher change in electrode potential (i. e., a higher overpotential).^[78]^ The internal resistance (*R*_int_) was also estimated as ratio between voltage and current at the maximum power (*P*_max_).

### COD and pH measurements

Liquid samples for COD measurement were collected at the beginning (after the electrolyte solution was prepared and inserted into the MFC) and at the end of each batch cycle (i. e., at days 7, 14, 21, 28, 35, and 42; Table S1). Each group utilized different colorimetric COD evaluation kits as reported in Table S2. In addition, pH was also measured at the beginning and end of each batch cycle. Each group utilized different pH‐meters as reported in Table S2.

### Microbial composition analysis

Each institution collected samples of the raw local wastewater, MFC influent (day 0 of each batch cycle), MFC effluent (day 7 of each batch cycle) and electrodes for microbial community analysis (Table S1). Influent and effluent samples were collected at six time‐points throughout the experiment. Biofilm samples from the electrodes (anodes and cathodes) were collected only at the end of the study (day 42). A total of 6 mL of well‐mixed raw wastewater was extracted and aliquots of 1 mL were inserted into 6 different microcentrifuge tubes. The tubes were centrifuged for 2 min at 10000 rpm at room temperature. The supernatant was removed from each tube and only the solid pellet containing the biomass remained on the bottom of the tube was kept for bacterial community analysis. The microcentrifuge tubes were then frozen at −80 °C until grouped shipment. The same procedure was applied for influent and effluent samples collected for each batch cycle and each MFC. At the end of the experiment, the anode and cathode electrodes of each MFC were submerged in liquid nitrogen and flash frozen. All samples were preserved at −80 °C until shipment.

Total genomic DNA was extracted from each sample by the J. Craig Venter Institute, La Jolla, CA, USA with the same method and tools using PowerBiofilm® DNA Isolation Kit (MO Bio, Carlsbad CA, P/N 24000–50). The DNA was used as a template to generate amplicons using primers 357F (5′‐CCTACGGGAGGCAGCAG‐3′) and 926R (5′‐CCGTCAATTCMTTTRAGT‐3′), targeted at the 16S rRNA locus and standard Illumina adapters. The amplicons were purified using PicoGreen assay (ThermoFisher Catalog no. P11496) according to manufacturer‘s instructions. Purified amplicons were sequenced using Illumina Miseq 2X150bp paired end technology. The raw sequencing output was checked for quality using FastQC. QIIME 1.0 was used to identify and remove chimeric sequences and perform quality trimming and taxonomy classification. The OTU table obtained was used to generate community profiles.

### Statistical analyses

Robust statistics was used for all data analyses. The use of Robust statistics was implied by the small number of replicates per group and by the variations in replicate responses that do not allow the use of Gaussian statistics.^[143]^ Robust statistic is also more suitable for analysis of data drawn from biological systems.^[144]^ Details related to the robust statistics are presented in the Supporting Information.

All data in this study are represented with their median ($\overset{↔}{x})$ and expanded uncertainty (*U*_exp_) in the form $\overset{↔}{x}\pm U_{exp}$ . Note that *U*_exp_, at a confidence level of approximately 95 %, is two times bigger than the standard deviation for a normal distribution.

The statistical significance between data sets was calculated using two‐sided Student's t‐test. Median and normalized median of absolute deviations (MADN) were used for the t‐test. Dixon's test was used to identify possible outliers. No outliers were identified. Principal component analysis (PCA) was used to analyze the data sets, find patterns and visualize correlations and anti‐correlations among samples and variables.[^145^, ^146^] PCA (XLSTAT Addinsoft) was applied to a dataset of samples collected from the influent and effluents throughout the study, and anodes and cathodes sampled after decommissioning of the reactors at the end of the experiment.

## Conflict of interest

The authors declare no conflict of interest.

## Supporting information

As a service to our authors and readers, this journal provides supporting information supplied by the authors. Such materials are peer reviewed and may be re‐organized for online delivery, but are not copy‐edited or typeset. Technical support issues arising from supporting information (other than missing files) should be addressed to the authors.

Supplementary
