# Supplementary material for: How Comparable are Microbial Electrochemical Systems around the Globe? An Electrochemical and Microbiological Cross‐Laboratory Study
Source: ChemSusChem. 2021 May 5;14(11):2313–30. doi: 10.1002/cssc.202100294 (PMC8252665; doi:10.1002/cssc.202100294)
Supplement: Supplementary file 1 — Supplementary [file CSSC-14-2313-s001.pdf]

ChemSusChem

Supporting Information

How Comparable are Microbial Electrochemical Systems around the Globe? An Electrochemical and Microbiological Cross-Laboratory Study

Carlo Santoro+,* Sofia Babanova+, Pierangela Cristiani+, Kateryna Artyushkova, Plamen Atanassov, Alain Bergel, Orianna Bretschger, Robert K. Brown, Kayla Carpenter, Alessandra Colombo#, Rachel Cortese, Benjamin Erable, Falk Harnisch, Mounika Kodali, Sujal Phadke, Sebastian Riedl, Luis F. M. Rosa, and Uwe Schröder© 2021 The Authors. ChemSusChem published by Wiley-VCH GmbH. This is an open access article under the terms of the Creative Commons Attribution License, which permits use, distribution and reproduction in any medium, provided the original work is properly cited.

Table of contents

Section 1. Abbreviations
Section 2. Voltage trend
Section 3. Polarization and power curves
Section 4. pH trend
Section 5. COD removal
Section 6. Coulombic Efficiency
Section 7. Microbiological Analysis
Section 8. Design of Experiments and Instruments utilized
Section 9. Statistical analysis

Section 1. Abbreviations

MFC –Microbial fuel cell
COD – Chemical oxygen demand
CE – Coulombic efficiency
UNM – University of New Mexico
RSE – Ricerca sul Sistema Energetico
CNR - CNRS, Université de Toulouse
TUB - Technische Universität Braunschweig
UFZ - Helmholtz-Centre for Environmental Research – UFZ
AC – Activated carbon
PTFE – polytetrafluoroethylene
KCl – potassium chloride
NaOH – sodium hydroxide
LSV- Linear sweep voltammetry
OCV – Open circuit voltage
I_{max} – maximum current
J_{max} – maximum current density
P_{max} – maximum power
V_{max} – maximum voltage
OCP – Open circuit potential
η_a – anode overpotentials
η_c – cathode overpotentials
OUT – Operational taxonomy unit
PCA – Principal components analysis
U_{exp} – expanded uncertainty
MAD – Median absolute deviation
EPS – esopolysaccarides

Section 2. Voltage trend

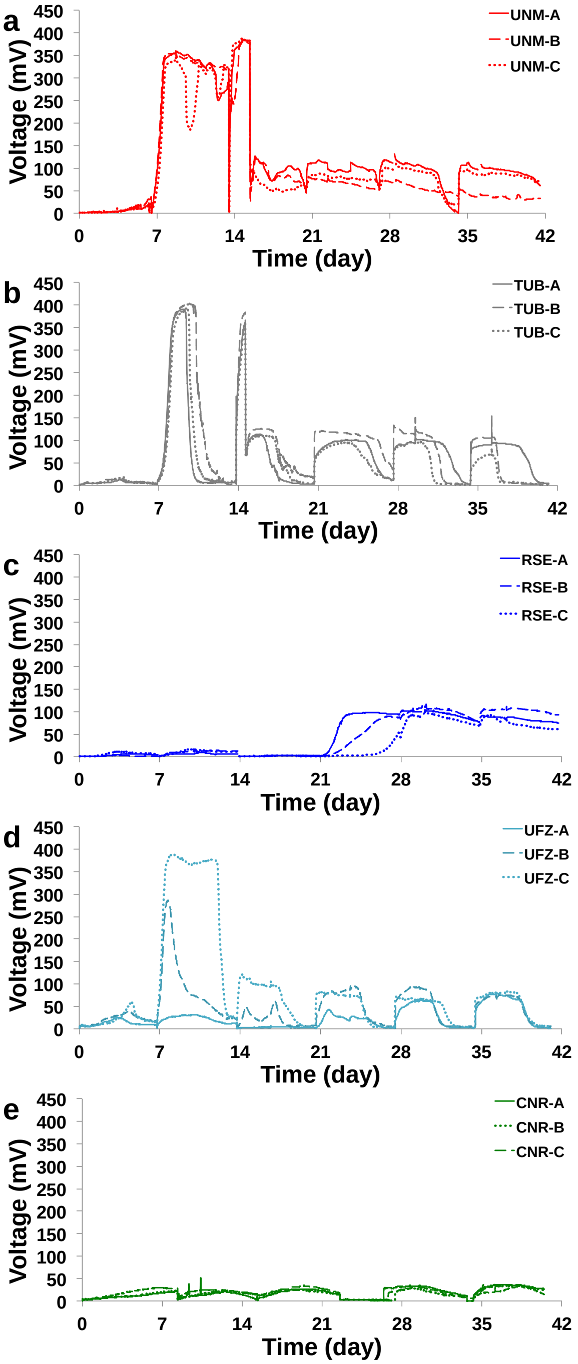

Figure S1. Voltage trend over time for the triplicate MFCs for each institution: UNM (a), TUB (b), RSE (c), UFZ (d) and CNR (e).

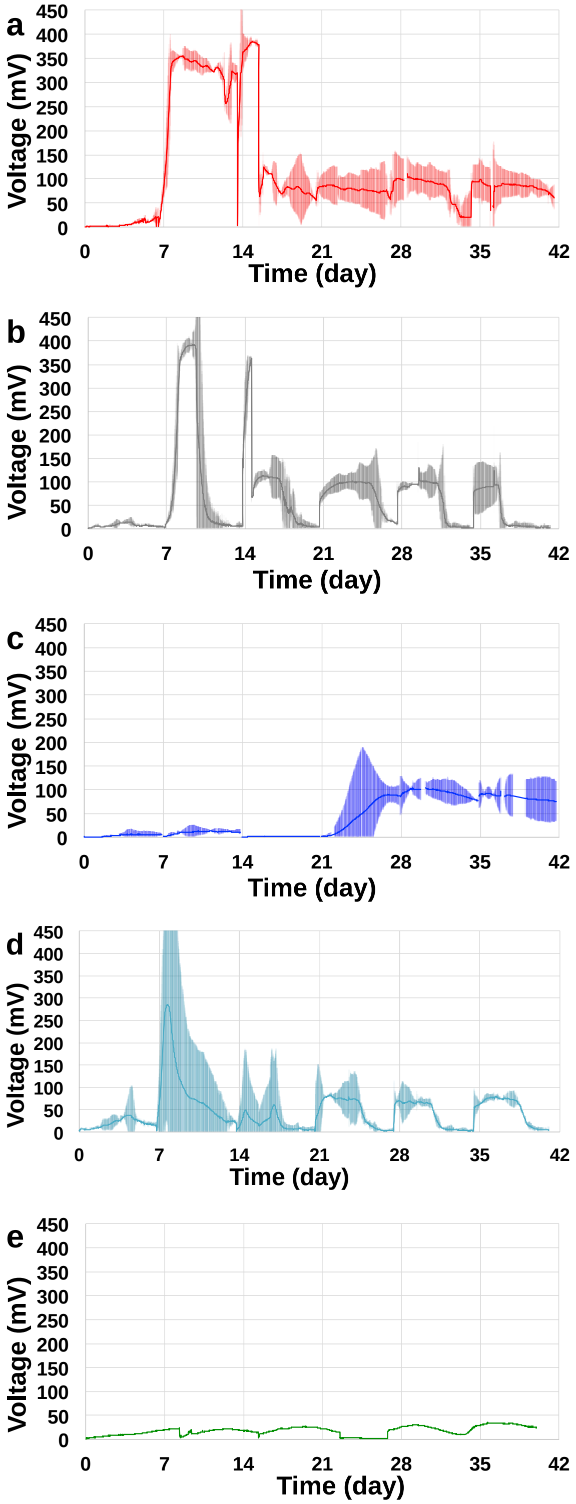

Figure S2. Voltage trend over time with U_{exp} for each institution: UNM (a), TUB (b), RSE (c), UFZ (d) and CNR (e).

Section 3. Polarization and power curves

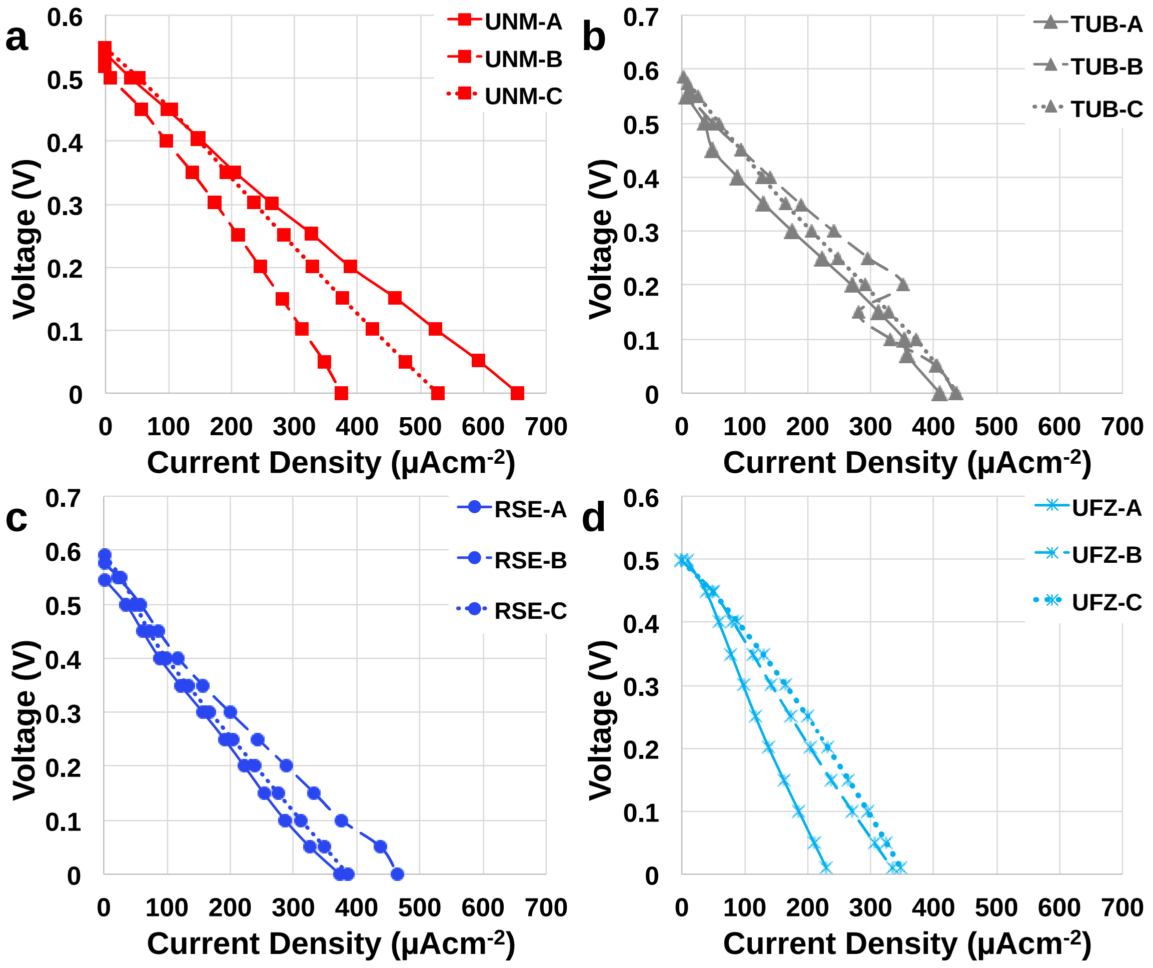

Figure S3. Polarization curves of all replicate MFCs measured separately during cycle 5: UNM (a), TUB (b), RSE (c), UFZ (d).

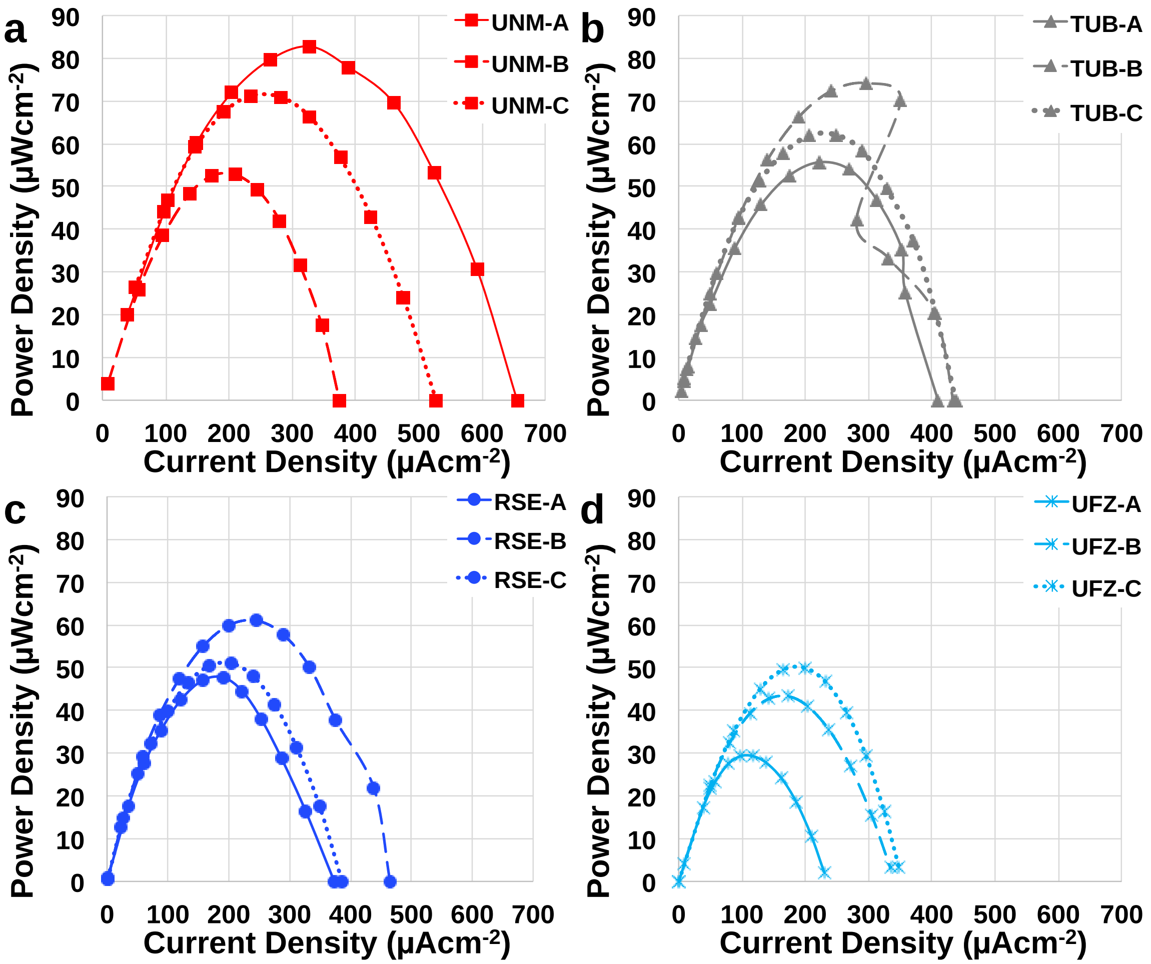

Figure S4. Power curves of all replicate MFCs measured separately during cycle 5: UNM (a), TUB (b), RSE (c), UFZ (d).

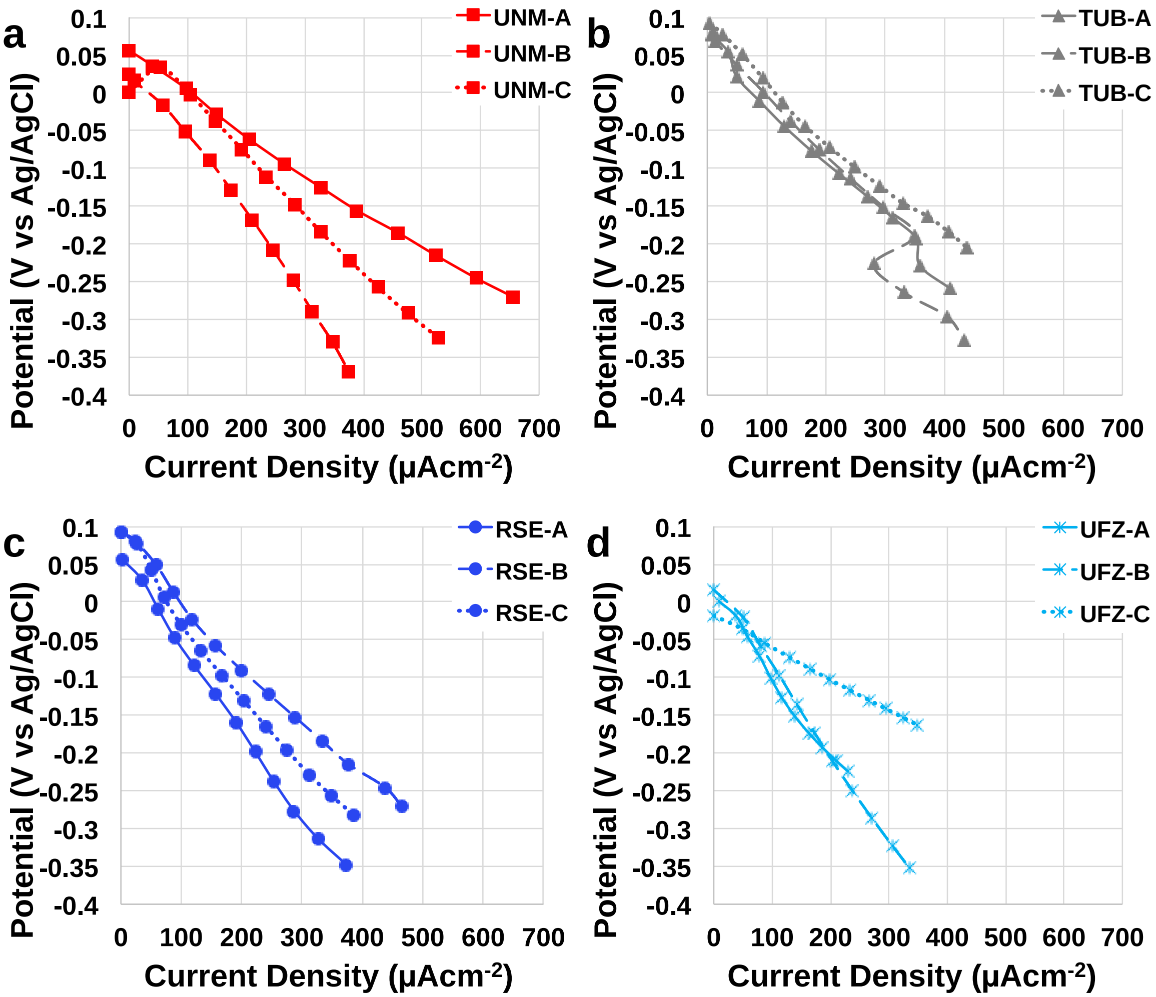

Figure S5. Cathode polarization curves of all replicate MFCs measured separately during cycle 5: UNM (a), TUB (b), RSE (c), UFZ (d).

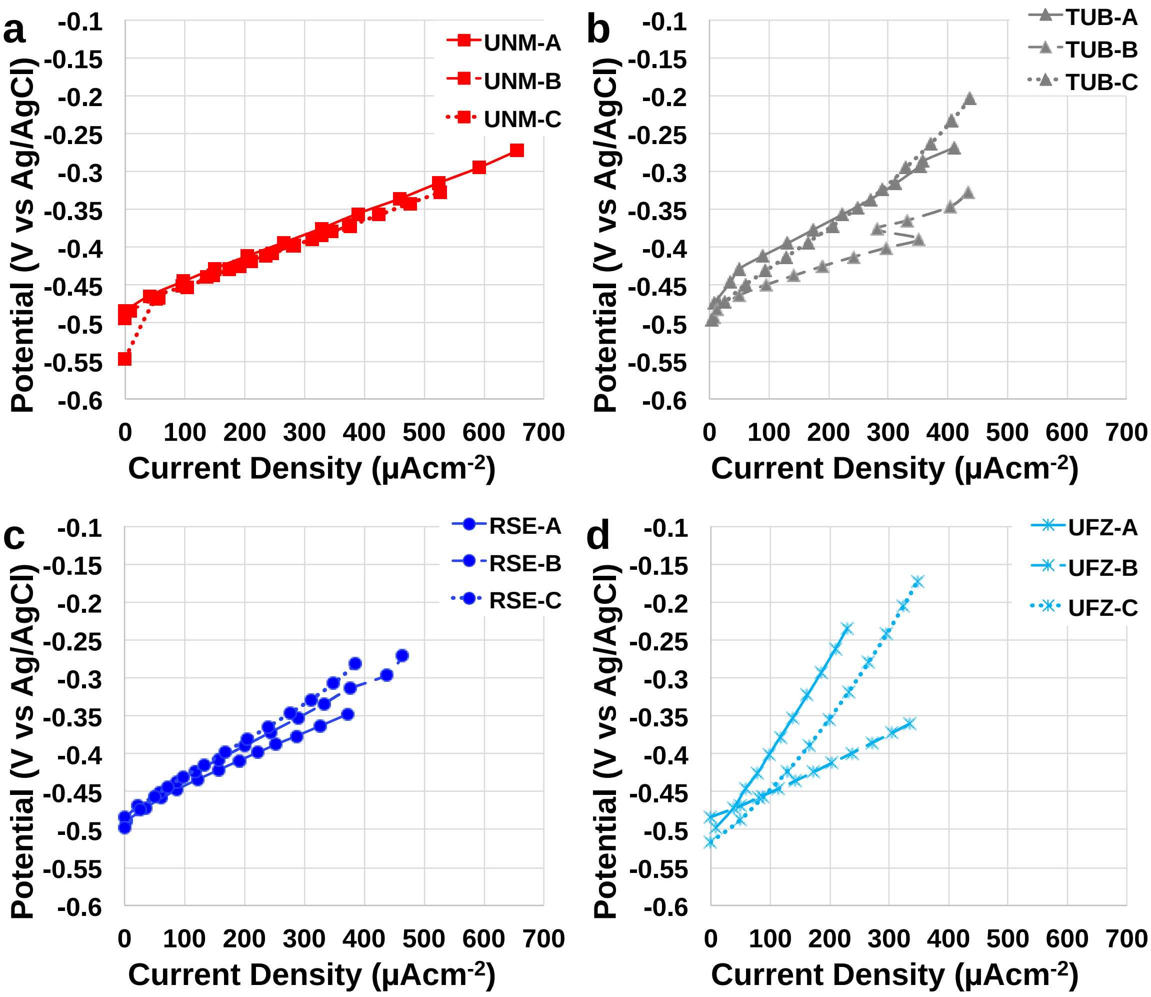

Figure S6. Anode polarization curves of all replicate MFCs measured separately during cycle 5: UNM (a), TUB (b), RSE (c), UFZ (d).

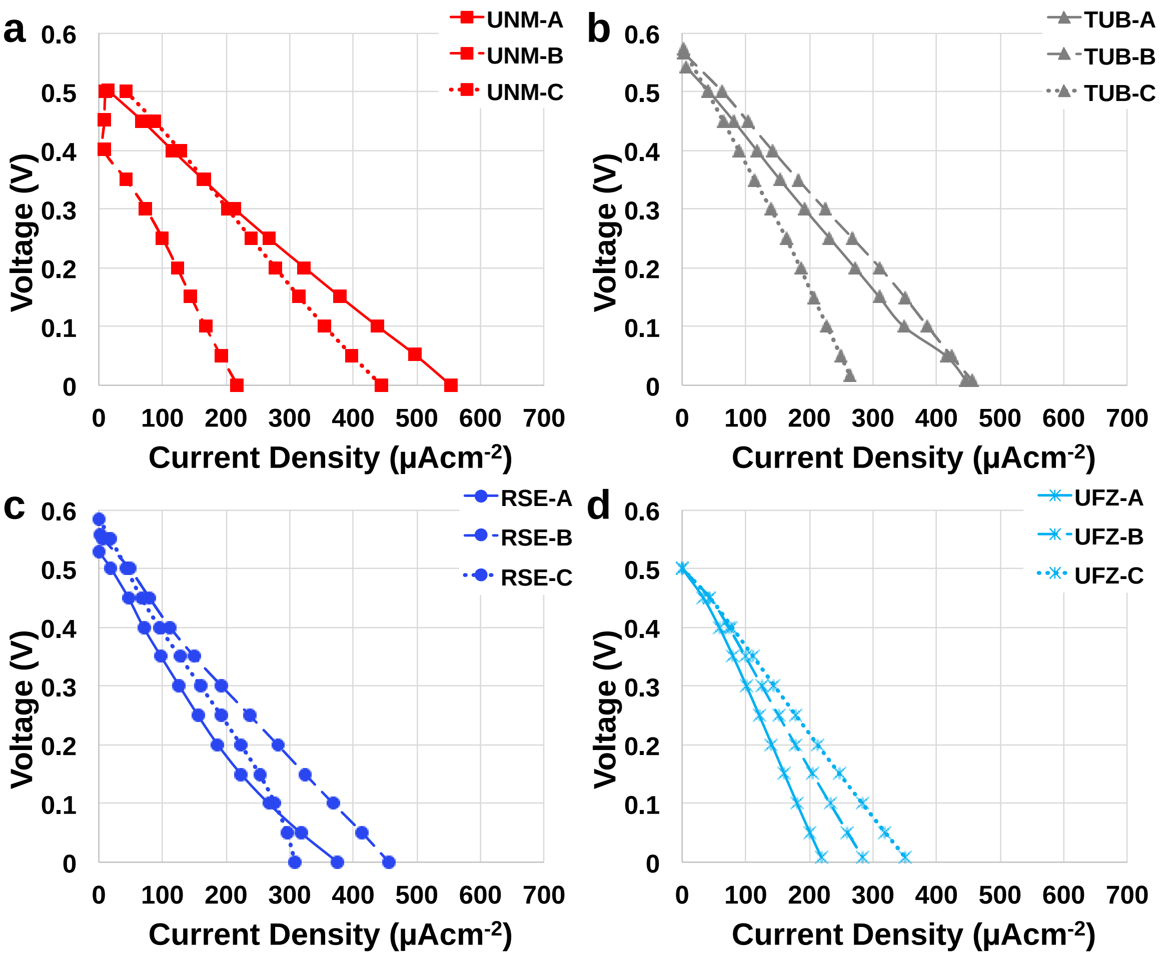

Figure S7. Polarization curves of all replicate MFCs measured separately during cycle 6: UNM (a), TUB (b), RSE (c), UFZ (d).

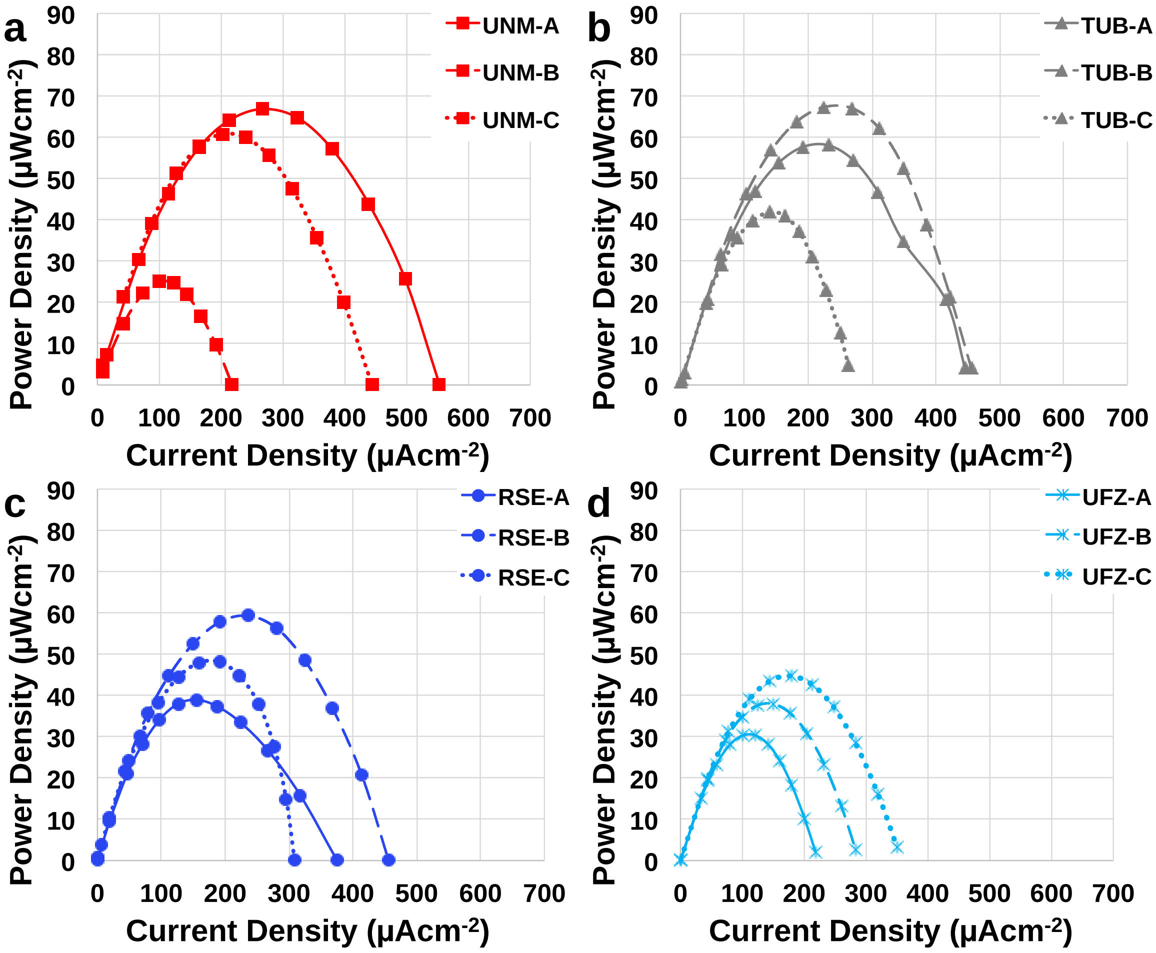

Figure S8. Power curves of all replicate MFCs measured separately during cycle 6: UNM (a), TUB (b), RSE (c), UFZ (d).

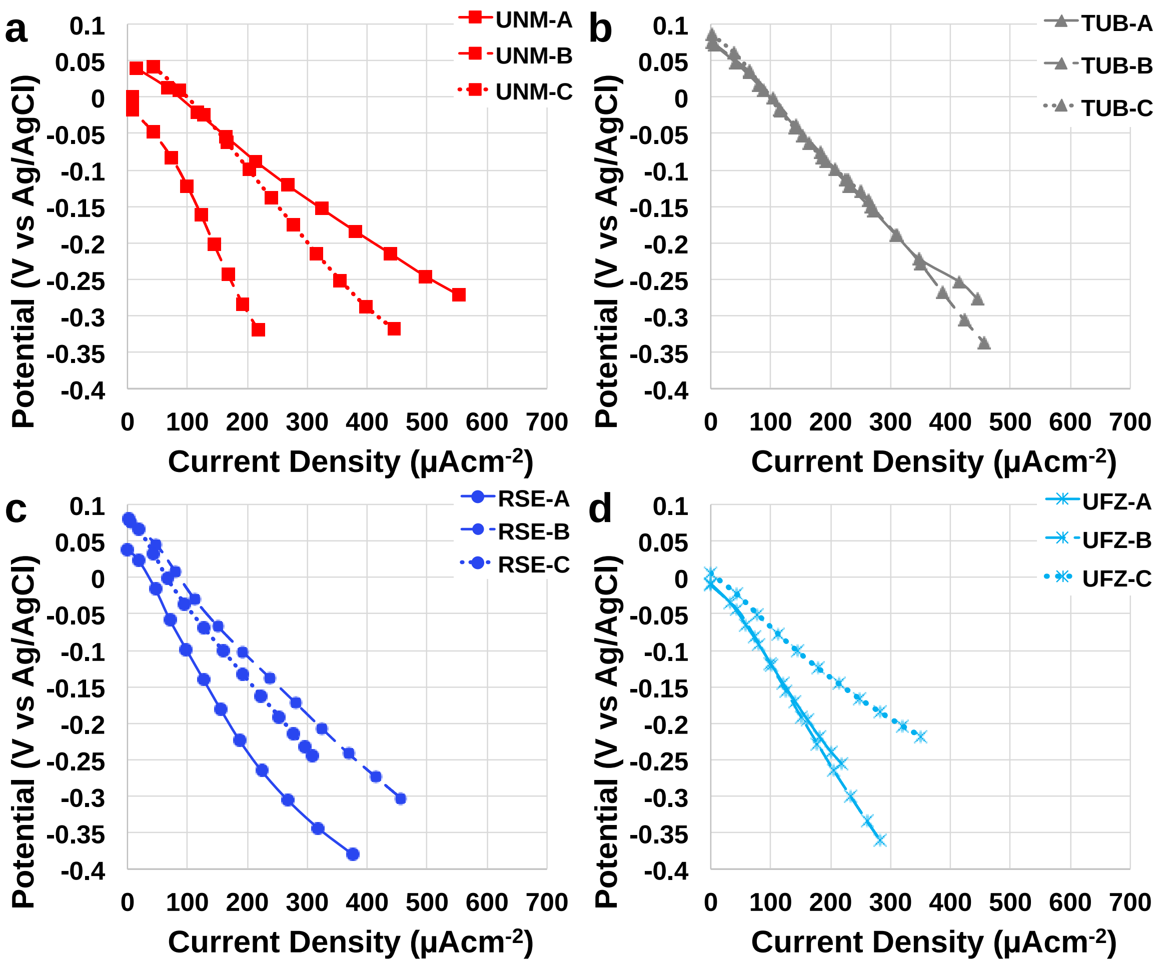

Figure S9. Cathode polarization curves of all replicate MFCs measured separately during cycle 6: UNM (a), TUB (b), RSE (c), UFZ (d).

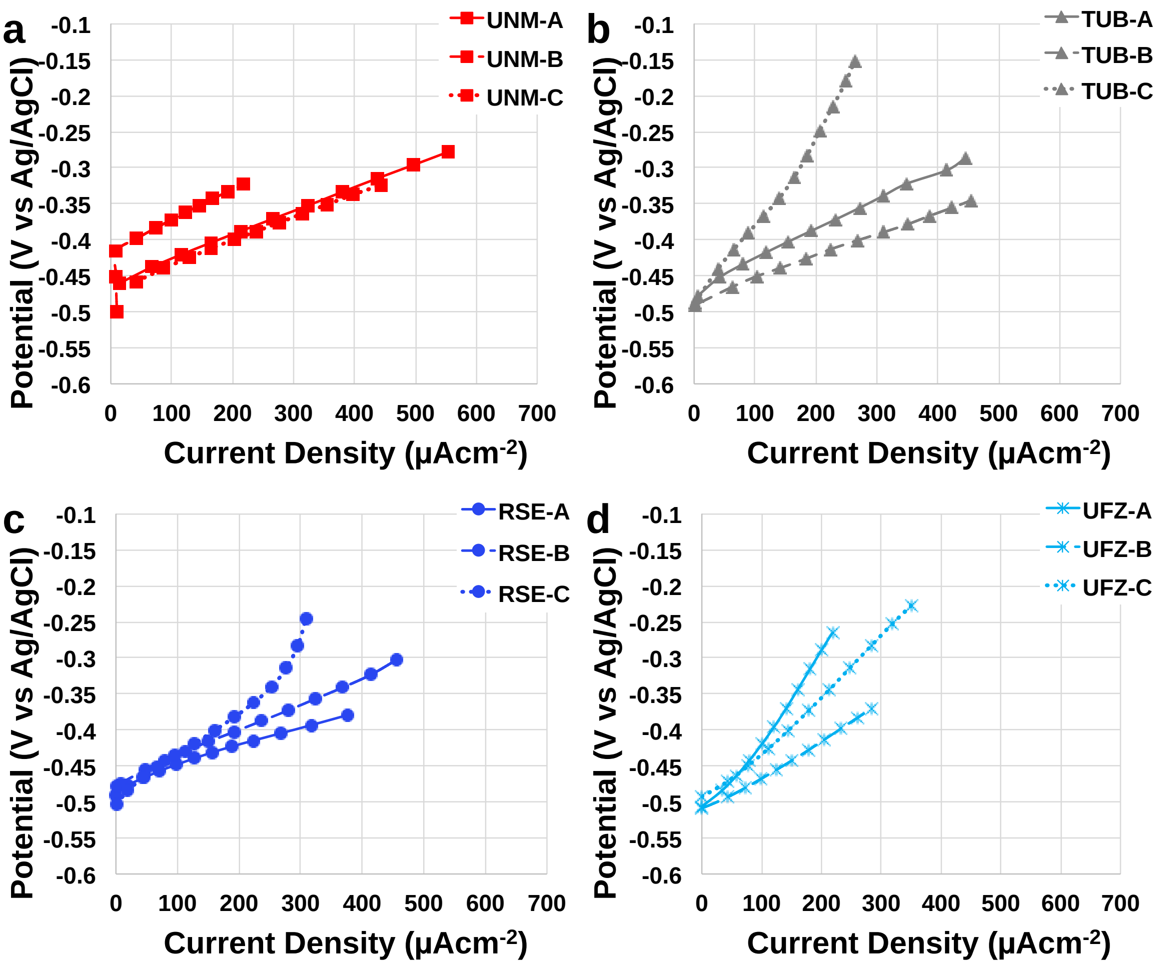

Figure S10. Anode polarization curves of all replicate MFCs measured separately during cycle 6: UNM (a), TUB (b), RSE (c), UFZ (d).

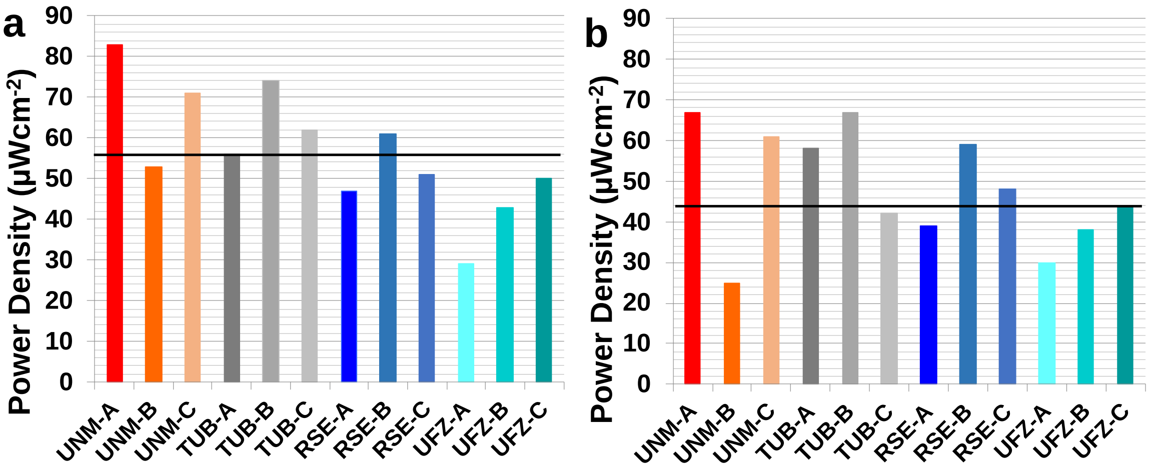

Figure S11. Power curve peak of all the MFCs measured separately during cycle 5 (a) and cycle 6 (b)

Section 4. pH trend

When it comes to electrochemical reactions involving protons, pH has an important role and it is one of the major factors affecting the reaction rate and efficiency. pH is even more important when biological species are involved in the process since their functionality is pH dependent. Oxygen reduction reaction (ORR) rate is pH dependent with higher kinetics at extreme pHs where H^+ and OH^- concentration is higher. At the same time, bacteria performing the anodic reaction require neutral pHs. pH below five or above 9-10 can have a significant impact on microbial population both in regard to fitness and diversity. Concerning the anode electrode, the maintenance of a constant, near neutral pH is a necessity.

The main reactions occurring in the MFCs are the following:

Anode reaction:

$$CH_3COOK + 2H_2O \rightarrow 2CO_2 + K^+ + 8e^- + 7H^+$$

Cathode Reaction:

$$4O_2 + 8e^- + 8H^+ \rightarrow 4H_2O_2$$

$$2O_2 + 8e^- + 8H^+ \rightarrow 4H_2O$$

Particularly, at the anode, acetate is oxidized and at the cathode, oxygen is reduced to water or hydrogen peroxide. During the oxidation of one mole of acetate, 7 moles of protons and 8 moles of electrons are produced, while during the reduction of oxygen 8 moles of protons and electrons are consumed for forming the final product. Therefore, there is a disbalance of protons that tends to be accumulated into the aqueous electrolyte and promote the pH variation.

In the first week, the final pH was identical for all institutions (Figure S12.a), pH~9, indicating a slight alkalization of the solution (Figure S12.b**)**. Interestingly, during the second week, UNM-MFCs had a drop in pH to 7.3±0.3 while the other 4 institutions still had high pH between 8.6 and 9.1. From week 3 to week 6, UNM-MFCs had a final pH that varied between 6.7 and 7.2. Also, the single UNM-MFCs did not show a great discrepancy during the last 3 cycles (Figure S13.a). The final pHs of TUB-MFCs decreased to 7.1±0.9 at cycle 4 and then increased back to 8.7±0.6 at cycle 5 and 8.4±0.3 at cycle 6. In the last two cycles, TUB-MFC-A had a final pH of 7.8 and 7.9 respectively while TUB-MFC-B and

-C had a final pH higher than 8.4 (Figure S13.b). RSE-MFCs had a more variable final pH that anyway tends to decrease with time. Particularly, at cycle 4 the final pH was 7.15±0.03, 7.0±0.3 in cycle 5 and 7.0±0.2 in the last cycle (Figure S13.c). The final pH of UFZ-MFCs stayed stable (~9.0) in the first 3 weeks and then gradually decreased to 8.7±0.3, still remaining within alkaline values (Figure S13.d). UFZ-MFCs final pH was very similar among the triplicate (Figure S13.d). CNR-MFCs had a constant pH between 8.9 and 9.1 all over the experimentation (Figure S13.e).

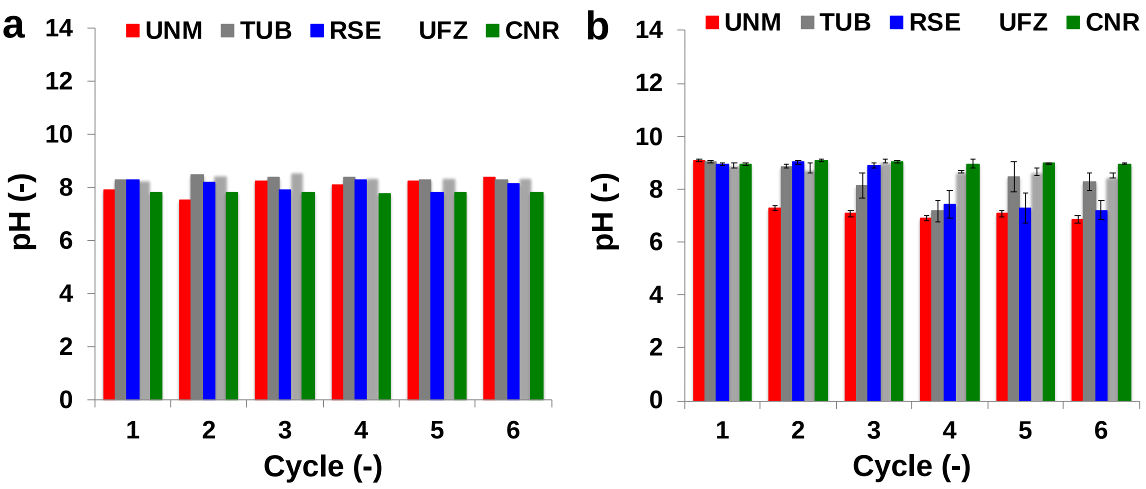

Figure S12. Initial (a) and final (b) solution pH. Each institution run triplicate experiments and the median with the expanded uncertainty are presented in the final solution pH.

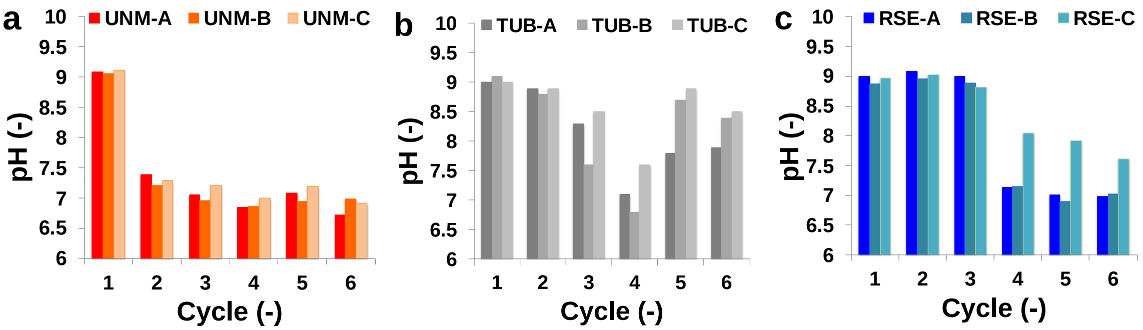

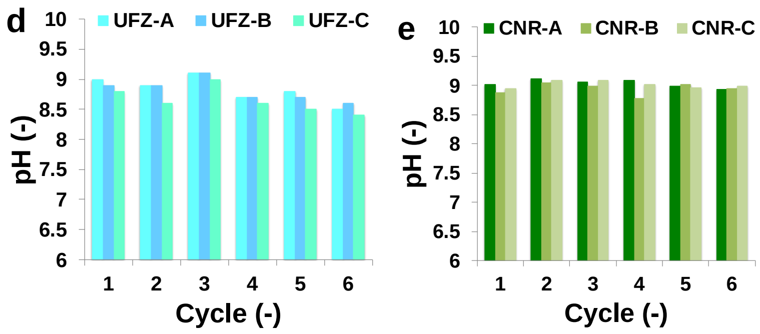

Figure S13. Final pHs of all the MFCs measured separately during the experimentation. UNM (a), TUB (b), RSE (c), UFZ (d), CNR (e).

Section 5. COD removal

Each institution prepared 0.7 L of solution and vigorously mixed before adding it to the triplicate MFCs. One sample was collected as representative of the initial COD loading for the triplicate MFCs (Figure S14.a). The medians of the final COD samples along with their expanded uncertainties are presented in Figure S14.b. The final COD samples for each MFC separately are presented in Figure S15. The COD values corresponding to each single MFC are presented in Figures S16-S17. For each institution separately, the initial COD (median of all cycles) was 1730±222 mgL^{-1} (U_{exp}=13%) for UNM, 1879±246 mgL^{-1} (U_{exp}=13%) for TUB, 1630±83 mgL^{-1} (U_{exp}=5%) for CNR, 1428±165 mgL^{-1} (U_{exp}=12%) for RSE and 1608±43 mgL^{-1} (U_{exp}=3%) for UFZ.

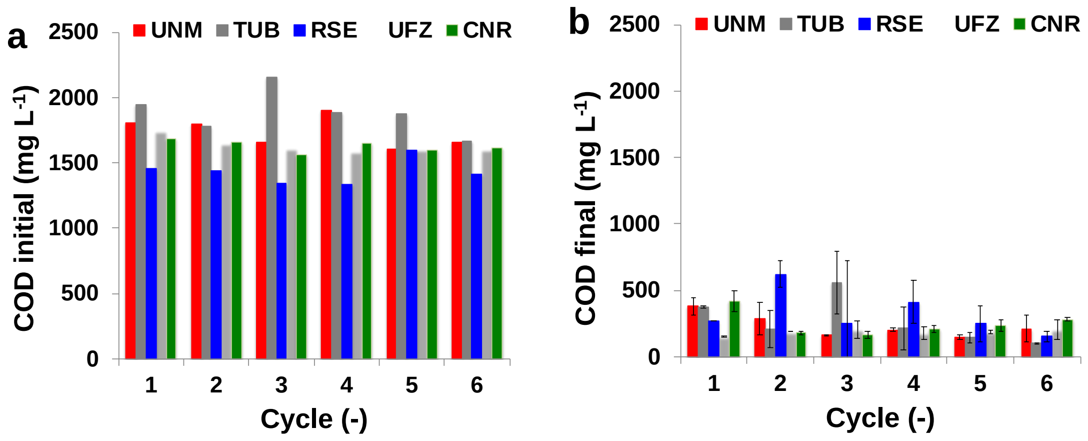

Figure S14. Initial and final COD concentrations for each institution during the six cycles.

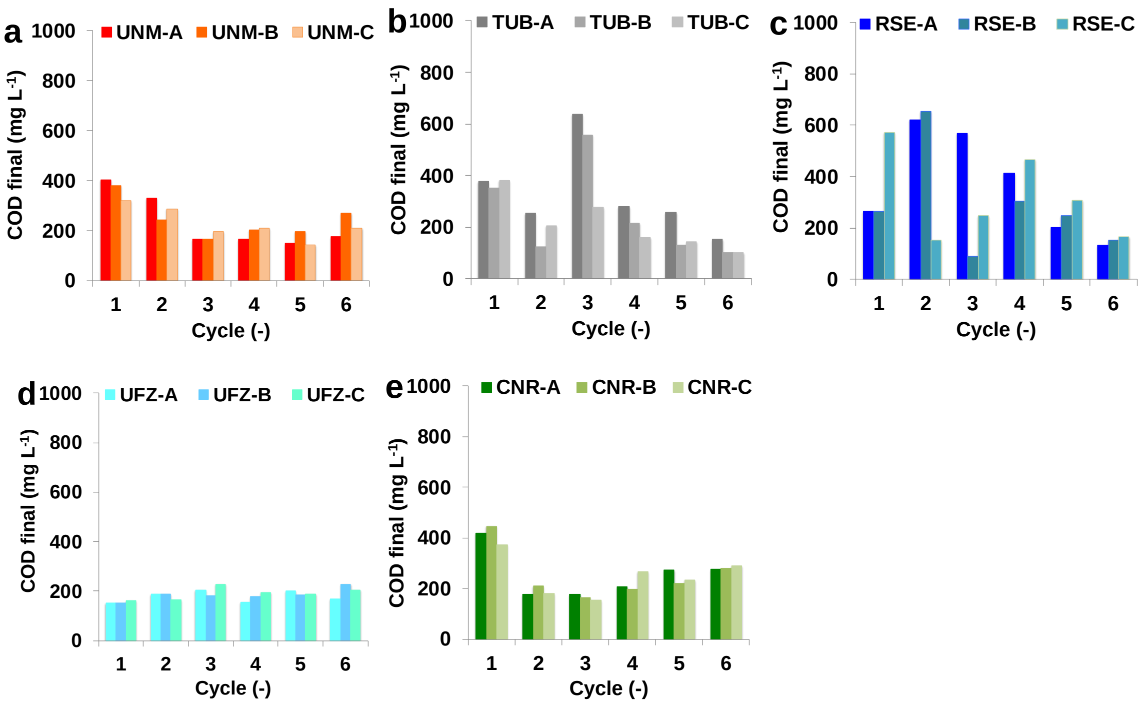

Figure S15. Final COD concentration of all replicate MFCs collected at the end of each cycle. UNM (a), TUB (b), RSE (c), UFZ (d), CNR (e).

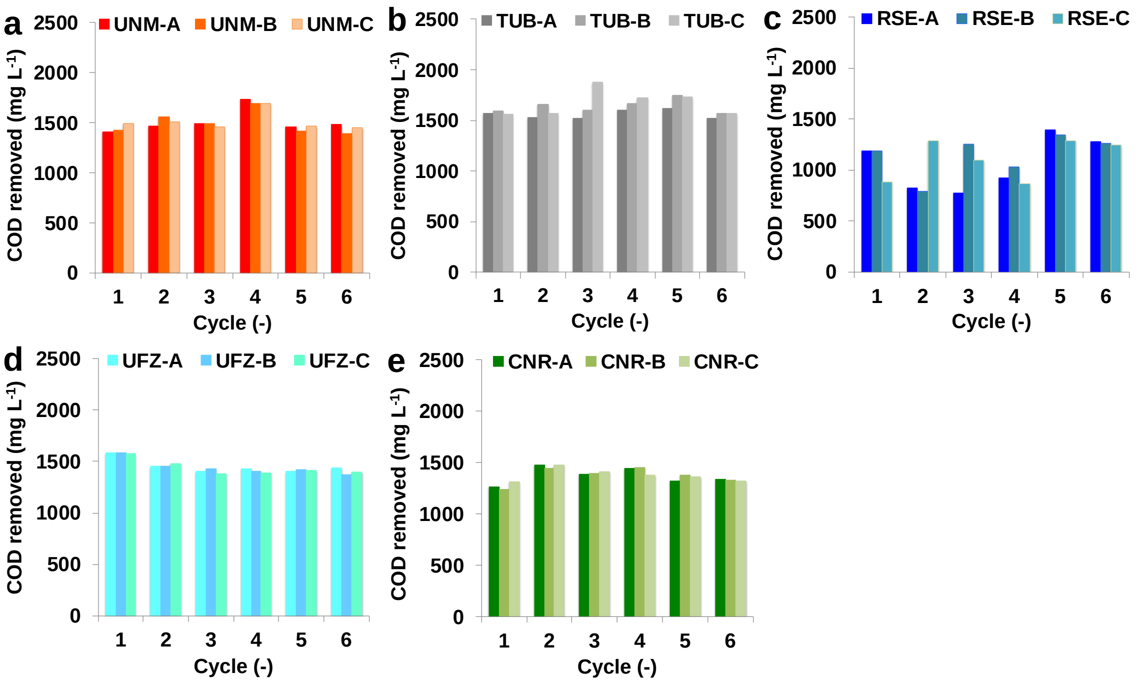

Figure S16. COD removed in terms of mgL^{-1} for all replicate MFCs measured at the end of each cycle. UNM (a), TUB (b), RSE (c), UFZ (d), CNR (e).

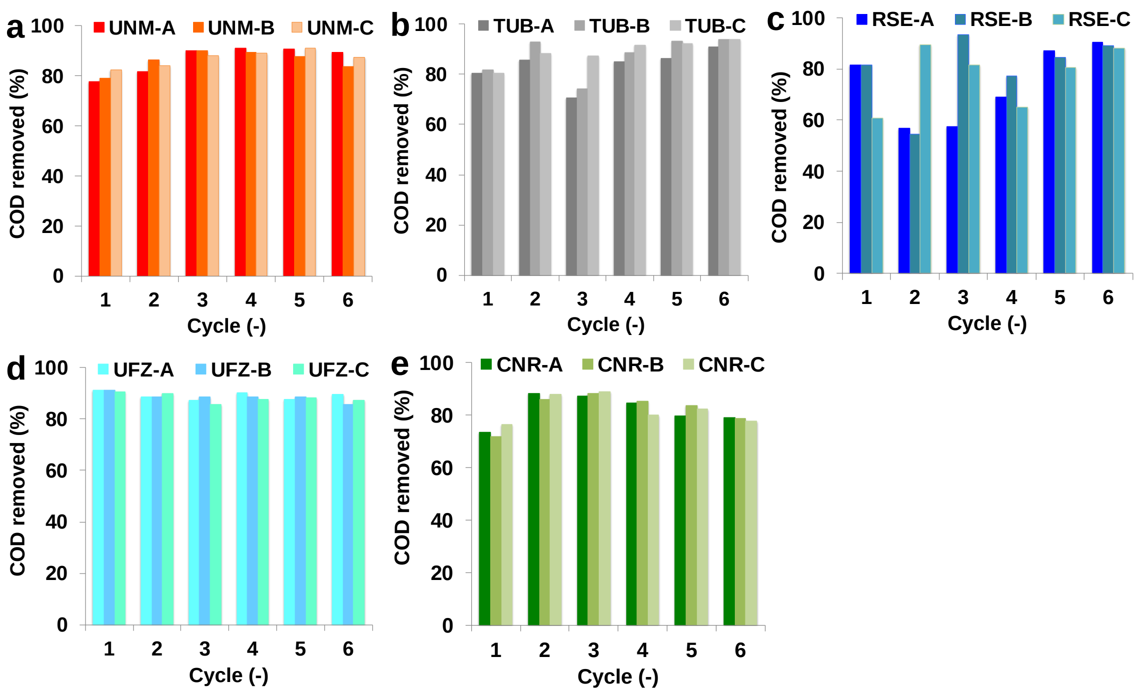

Figure S17. COD removed in terms of percentage for all replicate MFCs measured at the end of each cycle. UNM (a), TUB (b), RSE (c), UFZ (d), CNR (e).

Section 6. Coulombic Efficiency

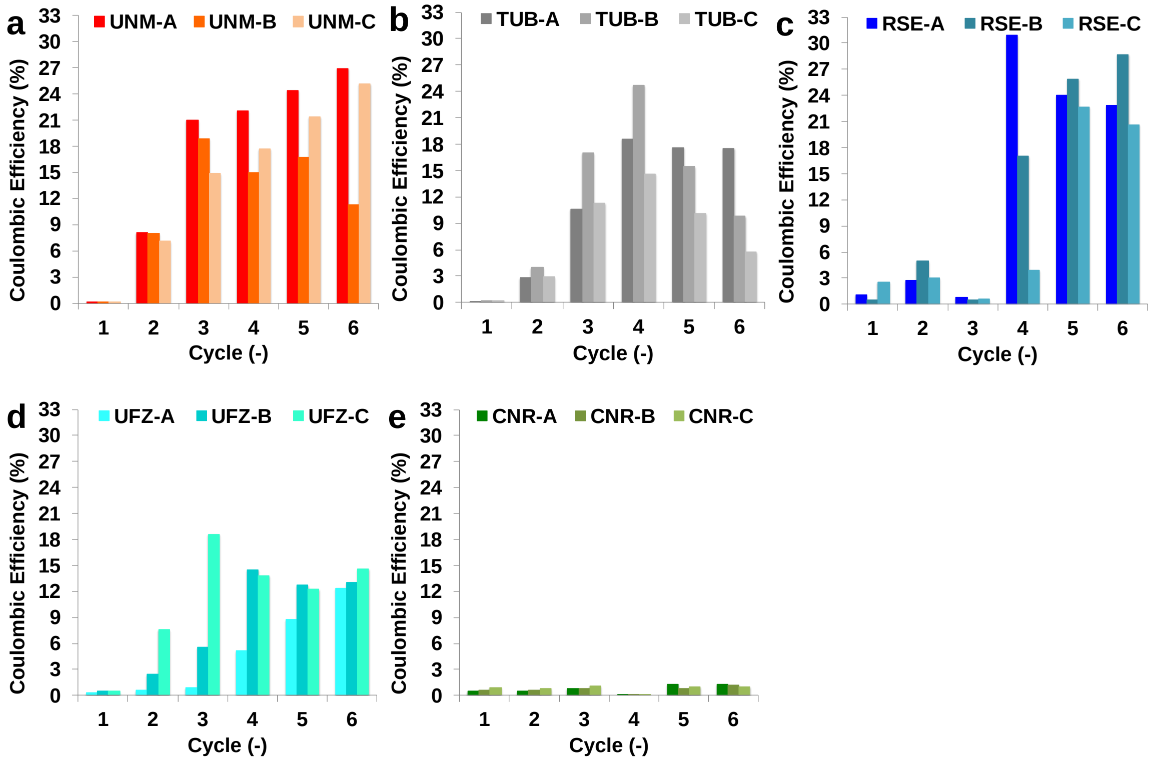

FIGURE S18. Coulombic efficiency of all replicate MFCs calculated for each cycle. UNM (a), TUB (b), RSE (c), UFZ (d), CNR (e)

Section 7. Microbiological Analysis

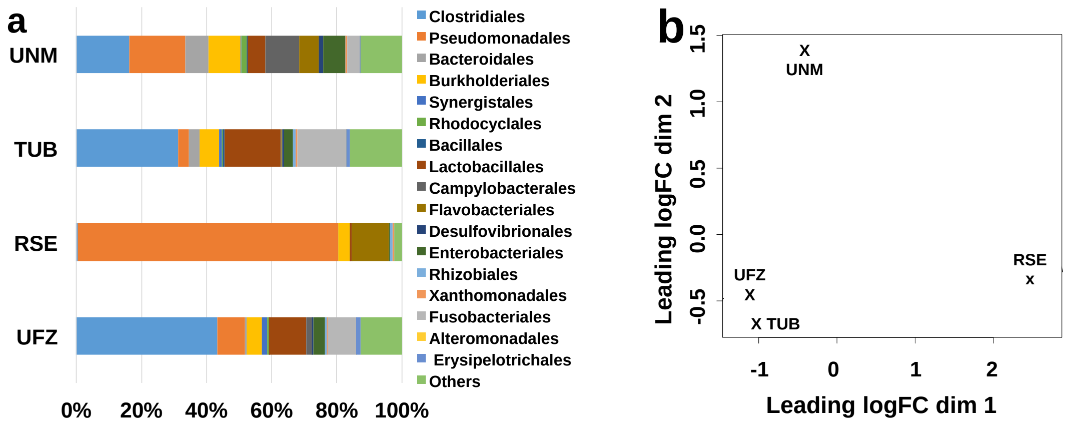

Figure S19. The community profiles at order level of the initial raw wastewater of the single research groups (a) and stress factor (b).

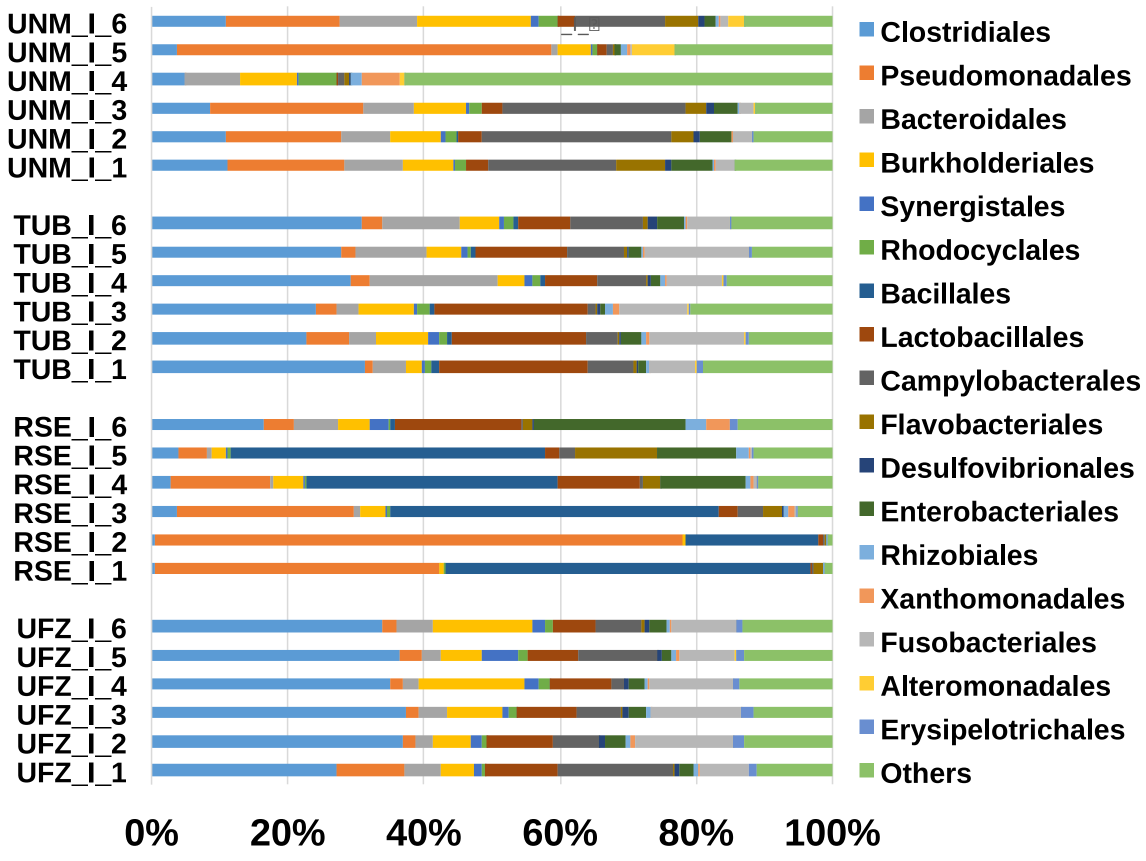

Figure S20. Order-level taxonomic distribution of 16S rRNA community profile within influent samples.

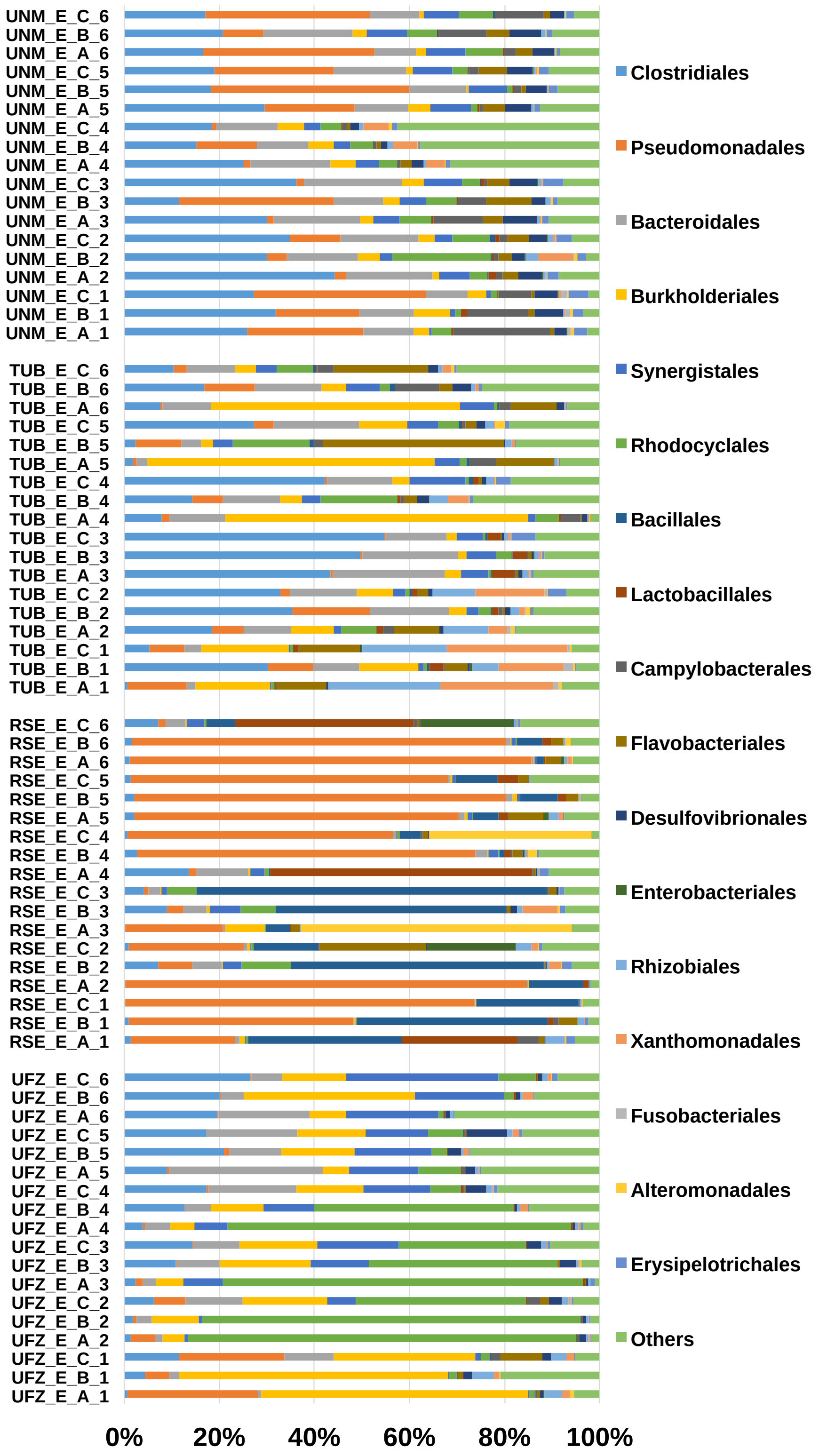

Figure S21. Order-level taxonomic distribution of 16S rRNA community profile within effluent samples.

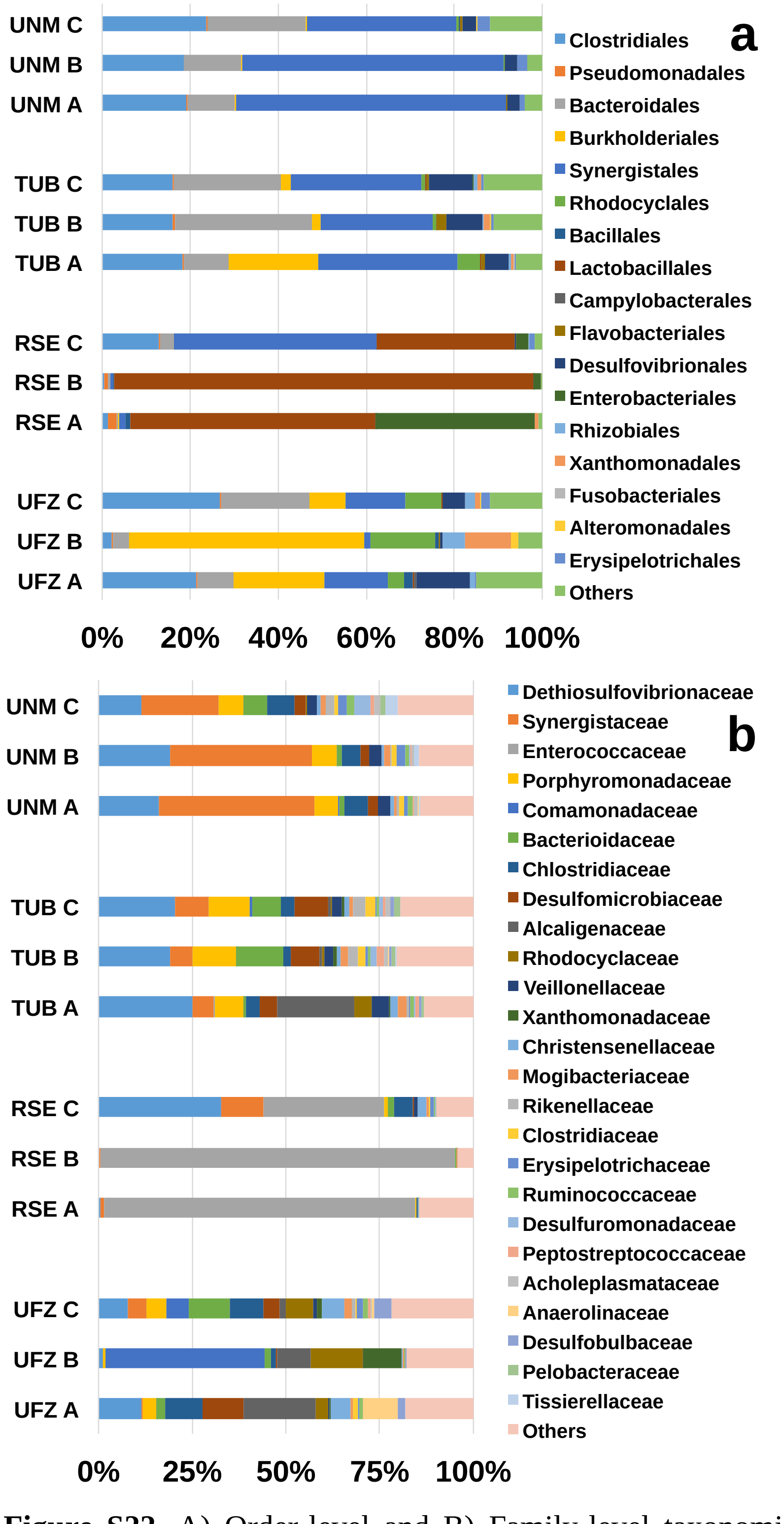

Figure S22. A) Order-level and B) Family-level taxonomic distribution of 16S rRNA community profile within anode electrochemically active biofilm.

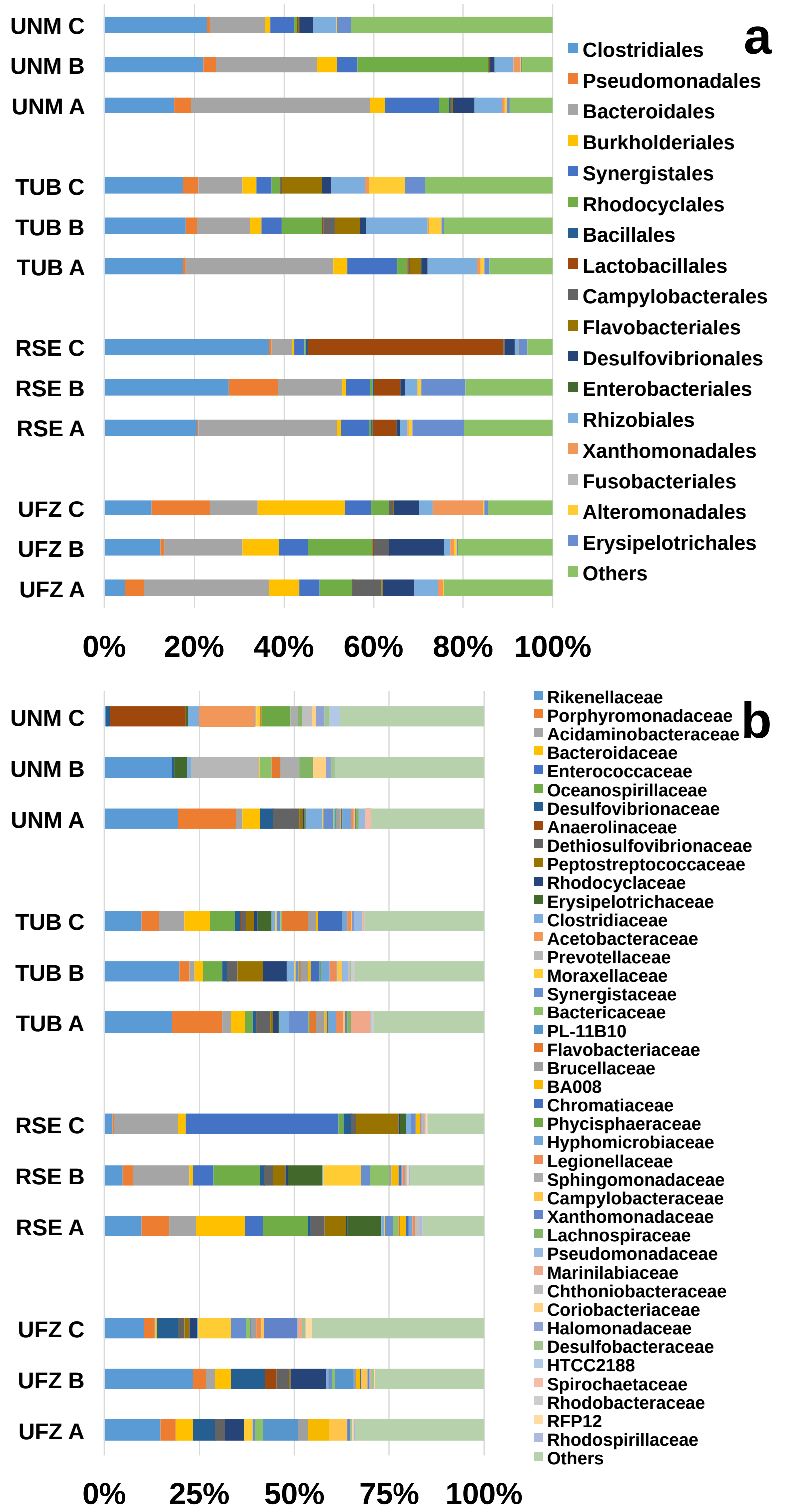

Figure S23. A) Order-level and B) Family-level taxonomic distribution of 16S rRNA community profile within cathode biofilm.

Section 8. Design of Experiments and Instruments utilized

Table S1. Design of Experiments: External resistance used, COD measurements, pH measurements and microbial sampling schedule.

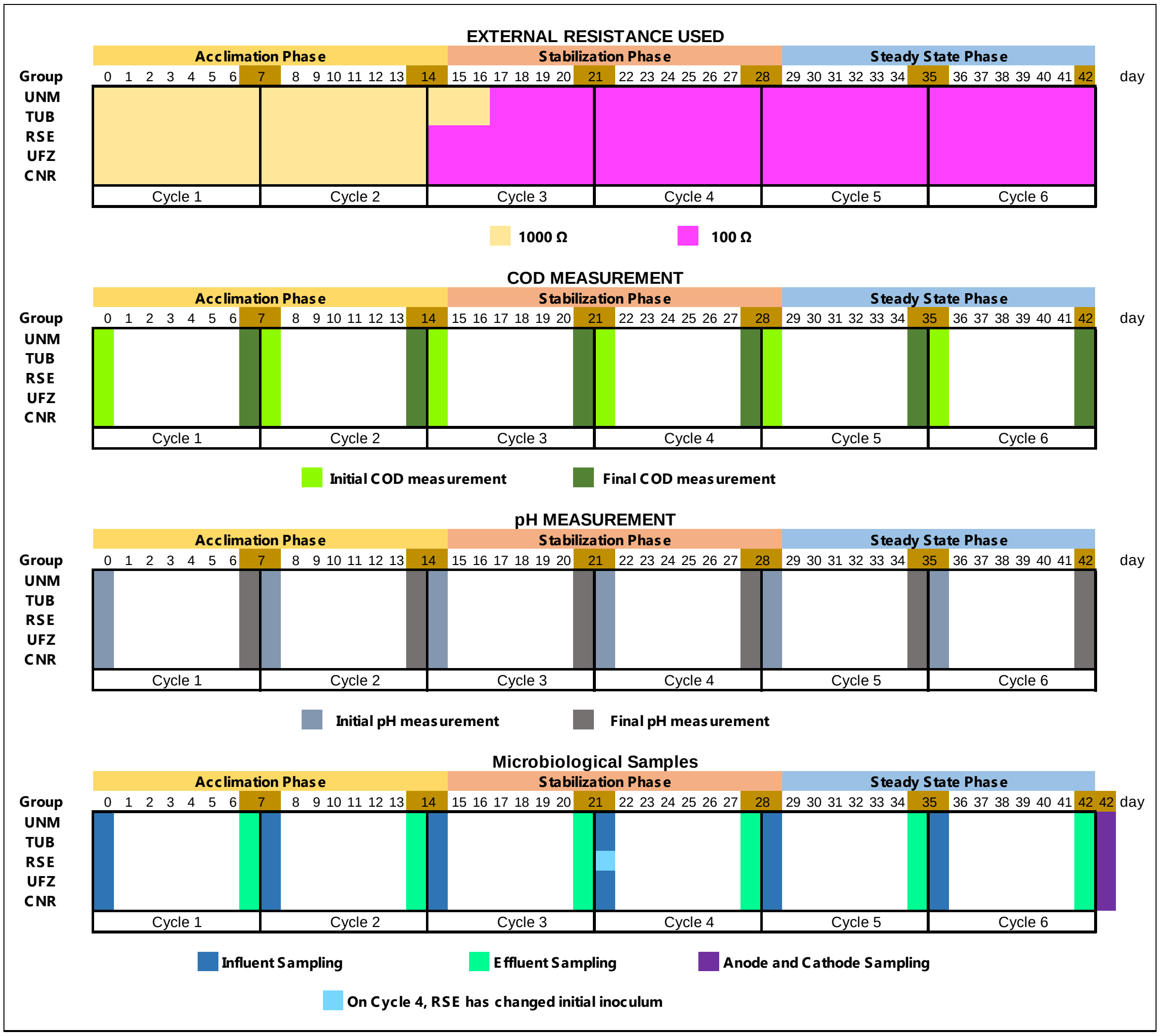

TABLE S2

Inoculum, potentiostats, data log system, COD measurement and pH-meter utilized during the experiments by each institution.

UNM team		
**Inoculum**	Albuquerque Southeast Water Reclamation Facility	Albuquerque, NM, USA
**Potentiostats**	Bio-Logic USA	Knoxville, TN, USA
**Data Log**	MCCDAQ Personal DAQ/56	Contoocook, NH, USA
**COD**	HACH standard Method	Loveland, CO, USA
**pH-meter**	Benchmeter OMEGA PHB-600R	Norwalk, CT, USA
**TUB team**		
**Inoculum**	Wastewater Treatment Plant Steinhof, Braunschweig	Braunschweig, Germany
**Potentiostats**	VMP3 (Bio-Logic Science Instruments)	Seyssinet-Pariset, France
**Data Log**	Digital multimeter Keithley 2701 via. Multiplexer 7700 (Tektronix)	Beaverton, OR, USA
**COD**	HACH standard Method	Düsseldorf, Germany
**pH-meter**	HI 2210 pH Meter (HANNA instruments)	Leighton Buzzard, UK
**RSE team**		
**Inoculum**	Milano-Nosedo wastewater treatment plant	Milano, Italy
**Potentiostats**	PAR / EG&G Model 273 Potentiostat / Galvanostat	Oak Ridge, TN, USA
**Data Log**	Graphtec midi Logger GL820	Yokohama, Japan
**COD**	HACH standard Method	Dusseldorf, Germany
**pH-meter**	pH-Meter AMEL 2235	Milano, Italy
**UFZ team**		
**Inoculum**	Abwasserzweckverband (AZV) für die Reinhaltung der Parthe	Am Klärwerk, 04451 Borsdorf, Germany
**Potentiostats**	Bio-Logic MPG 2	Bio-Logic, Seyssinet-Pariset, France
**Data Log**	Bio-Logic MPG 2	Bio-Logic, Seyssinet-Pariset, France
**COD**	HACH LCK 514 COD test (100 to 2000 mg L^{-1} COD	Düsseldorf, Germany
**pH-meter**	Hanna pH 201 pH meter	Germany
**CNR team**		
**Inoculum**	Castanet WWTP, SICOVAL	Castanet Tolosan, Occitanie, France
**Potentiostats**	VMP3	Bio-Logic, Seyssinet-Pariset, France
**Data Log**	VMP3	Bio-Logic, Seyssinet-Pariset, France
**COD**	HACH standard Method	Hach Lange France SAS, Villeurbane, France
**pH-meter**	SevenExcellence™ S700	Mettler Toledo SAS, Viroflay, France

Section 9. Statistical analysis

In Robust statistics, median of all replicates is an estimate of the measured value (Eq. S1).

$$\tilde{x} = median(x_i) \qquad \text{(Eq. S1)}$$

The normalized median of absolute deviations (MADN) in Robust statistics has the meaning of standard deviation in Gaussian statistics. MADN is calculated by dividing the median of absolute deviations (MAD (Eq. S2)) by 0.6745 (Eq. S3), where 0.6745 corresponds to MAD of a variable with normal distribution [1]. MADN is used to bridge data from Robust and Gaussian statistics, allowing data to be compared.

$$MAD = median(x_i - \tilde{x}) \qquad \text{(Eq. S2)}$$

$$MADN = MAD/0.6745 \qquad \text{(Eq. S3)}$$

Thus, the relative combined uncertainty in Robust statistics is calculated by Eq. S4 and the expanded uncertainty (U_{exp}) is estimated following Eq. S5:

$$U_c(y) = \frac{MADN}{\tilde{x}} \qquad \text{(Eq. S4)}$$

$$U_{exp} = k * U_c(y) \qquad \text{(Eq. S5)}$$

Where k is the coverage factor and for confidential level of approximately 95%, $k = 2$.
All data in this study are represented with their median and expanded uncertainty in the form $\tilde{x} \pm U_{exp}$.

Reference

[1] R.A. Maronna, R.D. Martin, V. J. Yohai C. Robust Statistics: Theory and Methods (2006) John Wiley & Sons, Ltd
